# CD73 activity controls cytotoxic CD4 T-cell response driving myocardial pathology in chronic Chagas disease

**DOI:** 10.3389/fimmu.2026.1770276

**Published:** 2026-05-12

**Authors:** Gastón Bergero, Yanina L. Mazzocco, Zoé M. Cejas Gallardo, Walter Rivarola, Sebastian Del Rosso, Maria P. Aoki

**Affiliations:** 1 Universidad Nacional de Córdoba, Facultad de Ciencias Químicas, Departamento de Bioquímica Clínica, Córdoba, Argentina; 2 Consejo Nacional de Investigaciones Científicas y Técnicas (CONICET), Centro de Investigación en Bioquímica Clínica e Inmunología (CIBICI), Córdoba, Argentina; 3 Universidad Nacional de Córdoba, Facultad de Ciencias Médicas, Centro de Investigación de la Enfermedad de Chagas y Leishmaniasis, Córdoba, Argentina; 4 Consejo Nacional de Investigaciones Científicas y Técnicas (CONICET), Instituto de Investigaciones en Ciencias de la Salud (INICSA), Córdoba, Argentina

**Keywords:** adenosine, cardiomyopathy, CD4 CTL, granzyme B, *Trypanosoma cruzi* infection, immunoregulation, cardiac immune response, ectonucleotidases

## Abstract

Chagas disease, caused by *Trypanosoma cruzi*, is the major cause of infectious cardiopathology worldwide. Although cytotoxic CD4 T-cells (CD4 CTLs) have recently been recognized as crucial effectors in infections and inflammation, the mechanisms that control their differentiation and impact on pathological outcomes remain largely undefined. Here, we demonstrate that the ectonucleotidase CD73, which generates adenosine from extracellular AMP, acts as a key immunoregulator of CD4 CTL response during *T. cruzi* infection. Using murine models, we found that infection induced a robust expansion of CD4 T-cells expressing granzyme B, perforin, and IFN-γ. CD73 deficiency improved parasite control and amplified the frequency and cytotoxic program of CD4 T-cells during the acute phase. However, the absence of CD73 also led to sustained cardiac inflammation, extensive fibrosis, and impaired contractility during chronic infection. In patients with asymptomatic chronic Chagas disease, circulating CD4 T-cells exhibited elevated granzyme B expression, predominantly within the CD73^-^ subset. Consistently, cardiac tissue from patients with chronic terminal Chagas cardiomyopathy showed transcriptomic enrichment of granzyme B (*GZMB*), perforin (*PRF1*), and IFN-γ (*IFNG*), with CD4 T-cells as the major contributors. Together, these findings identify CD73 ectoenzyme as a critical immunometabolic checkpoint that modulates CD4 CTL responses, revealing a dual role for this pathway in controlling infection and limiting tissue damage.

## 1 Introduction

Chagas disease, caused by the intracellular parasite *Trypanosoma cruzi*, remains one of the major neglected tropical diseases in Latin America and an emerging global health problem due to migration. Indeed, recent evidence has led experts to propose that the United States should now be considered an endemic country for Chagas disease (1). About 30% of infected individuals will develop, several decades after the primary infection, chronic Chagas cardiomyopathy (CCC), the leading cause of infectious myocarditis worldwide (2). Studies of CCC pathogenesis have significantly deepened our knowledge of cardiac immunobiology; however, key mechanisms are still not fully understood. Cell-mediated immunity, mainly orchestrated by T-cells and macrophages, controls parasite replication but fails to achieve sterilizing immunity (3), allowing a persistent low-level parasite burden that sustains chronic myocardial inflammation and progressive pathological tissue remodeling (4). Although T-cell-mediated cytotoxicity has been traditionally attributed to the CD8 T-cell compartment, a subset of CD4 T-cells with cytotoxic potential (CD4 CTLs) has emerged as a mediator of host protection in viral infections, tumors, and, more recently, in Chagas disease (5–7). This cell population is characterized by its ability to secrete IFN-γ and to release cytotoxic granules containing granzyme B and perforin, which directly kill MHC class II-bearing target cells. Nevertheless, accumulating evidence also implicates CD4 CTLs in tissue injury (8, 9), highlighting their dual role in host defense and immunopathology. Given that the molecular cues governing their differentiation and function remain poorly defined (10), it is critical to elucidate the signals required for their development.

One key immune-regulatory system in inflamed tissues is the purinergic signaling pathway. Damaged tissue and activated leukocytes release into the extracellular space large amounts of ATP, which exerts pro-inflammatory and microbicidal effects through P2 receptors (R), mainly P2X7R (11). The half-life of ATP is short because CD39 and CD73 (ecto-5′-nucleotidase) ectonucleotidases sequentially hydrolyze it. CD39 converts ATP/ADP to AMP, and CD73 degrades AMP to adenosine (ADO) (12). ADO, in turn, dampens excessive inflammation and promotes tissue repair mechanisms through activation of A2a and A2b receptors (13). In this context, purinergic signaling has emerged as a critical regulator of the anti-*T. cruzi* immune response, limiting tissue damage but at the same time favoring parasite persistence (6, 14, 15).

CD73 plays a pivotal role in modulating the balance between ATP-driven pro-inflammatory signaling and ADO-mediated immunosuppression. While co-expression of CD39 and CD73 is a hallmark of FoxP3^+^ regulatory T-cells (16), a CD39^+^CD73^-^ phenotype has been associated with effector control responses in non-regulatory T-cells (6, 17–19). In experimental models, we previously demonstrated that early pharmacological inhibition of CD73 after *T. cruzi* infection enhances the cardiac T-cell response and delays the onset of Chagas cardiomyopathy (14). Moreover, transcriptomic analyses revealed that cardiac tissues from patients with end-stage CCC are enriched in CD73 transcripts (6), and that CD73 expression in infiltrating cardiac leukocytes, predominantly T-cells, correlates with both local parasite load and disease severity (20). These findings suggest that CD73 activity critically influences T-cell function and the outcome of Chagas disease.

In the present study, we comprehensively investigated the role of CD73 activity in regulating CD4 CTLs during *T. cruzi* infection. Our findings demonstrated that inhibition of CD73 activity specifically enhances the expansion of cytotoxic CD4 but not CD8 T-cells, and improves parasite control during the acute phase of infection. Unexpectedly, this heightened response was also associated with functional heart alterations and increased cardiac tissue injury during the chronic phase, suggesting that CD4 CTLs may be protective during the early phase but potentially immunopathogenic at later stages of infection. In agreement, patients with chronic Chagas disease showed increased frequency of CD4 T-cells with cytotoxic potential in both peripheral blood and cardiac tissue. Collectively, these findings highlight the pivotal role of CD73 activity in influencing CD4 CTL differentiation and function during *T. cruzi* infection and underscore purinergic signaling as a potential therapeutic target to enhance protective immunity without compromising tissue integrity in patients with Chagas disease.

## 2 Results

### 2.1 *T. cruzi* infection expands a population of CD4 T-cells with cytotoxic potential

**Figure 1:**
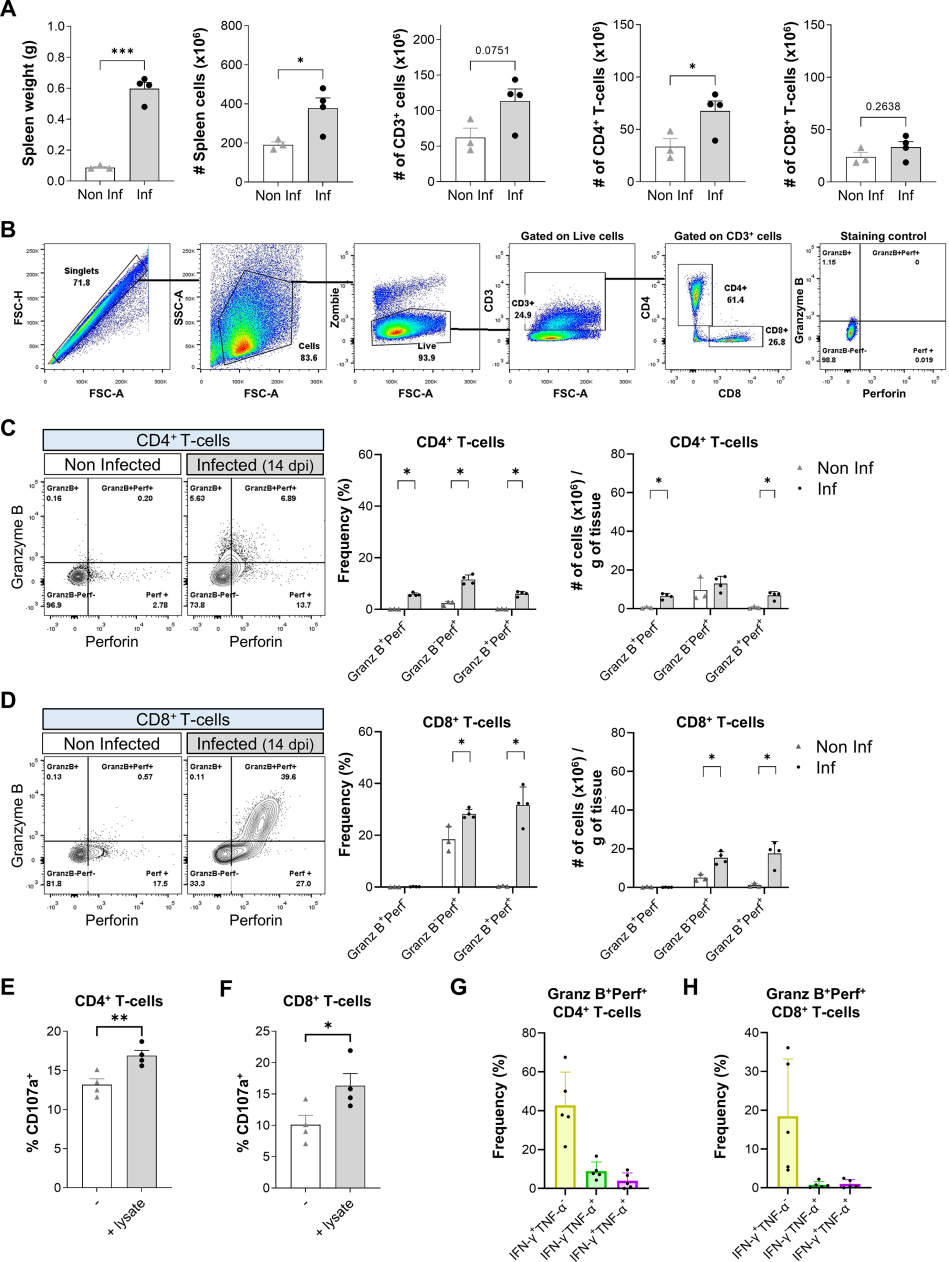
Expansion of cytotoxic CD4 T-cells during acute *T. cruzi* infection. **(A)** Spleen weights and total numbers of splenic cells, CD3^+^ cells (T-cells), CD4^+^ and CD8^+^ T-cells from non-infected (Non Inf, n = 3) and infected mice at 14 dpi (Inf, n = 4). **(B)** Gating strategy to identify splenic T-cells with cytotoxic phenotype by flow cytometry. **(C, D)** Representative contour plots, frequencies and number of cells per gram of tissue of GranzB^+^Perf^-^, GranzB^-^Perf^+^, and GranzB^+^Perf^+^ cells in CD4^+^ **(C)** or CD8^+^ **(D)** T-cells in spleens from non-infected mice and at 14 dpi. **(E, F)** Frequencies of CD107a^+^ in CD4^+^ **(E)** or CD8^+^ **(F)** T-cells in spleens from infected mice (14 dpi, n = 4) after *T. cruzi* lysate stimulation. **(G, H)** Frequencies of IFN-γ^+^TNF-α^-^, IFN-γ^-^TNF-α^+^ and IFN-γ^+^TNF-α^+^ in GranzB^+^Perf^+^CD4^+^ **(G)** or GranzB^+^Perf^+^CD8^+^ **(H)** T-cells in spleens at 14 dpi (n = 4) after PMA/ionomycin stimulation. Independent samples t-tests were performed to compare Non Inf vs. Inf. *p < 0.05, **p < 0.01, ***p < 0.001.

First, we sought to investigate the development of T-cell-mediated cytotoxicity during acute *T. cruzi* infection. At 14 days post-infection (dpi), *T. cruzi* infection significantly increased spleen weight and total splenic cellularity compared with non-infected controls, indicating a robust splenic expansion in response to infection. Notably, the absolute number of CD4^+^ T-cells, but not CD8^+^ T-cells, significantly increased after infection (**Figure 1A**). Following the gating strategy illustrated in **Figure 1B**, we found that infected WT mice exhibited a significant increase in both the frequencies and counts per grams of tissue of splenic CD4 T-cells co-expressing granzyme B and perforin (**Figure 1C**). In parallel, a concomitant expansion of cytotoxic CD8 T-cells was also observed (**Figure 1D**).

To determine whether these cytotoxic populations were antigen-dependent, splenocytes from infected mice were restimulated ex vivo with *T. cruzi* lysate. Upon antigenic restimulation, both CD4 and CD8 T-cells exhibited increased expression of the degranulation marker CD107a (**Figures 1E, F**), indicating that the expanded cytotoxic T-cell populations depend on parasite antigens.

Notably, approximately 40% of GranzB^+^Perf^+^ CD4 T-cells concomitantly produced IFN-γ, whereas TNF-α expression remained comparatively low (**Figure 1G**). The results indicate that CD4 CTLs display a multifunctional effector program combining cytotoxicity with type 1 inflammatory functions. Although cytotoxic CD8 T-cells reached higher absolute numbers than CD4 CTLs, their ability to produce IFN-γ and TNF-α was lower than that of CD4 CTLs (**Figure 1H**), highlighting the distinct functional potential of CD4 versus CD8 cytotoxic subsets during infection.

### 2.2 CD73 deficiency promotes the cytotoxic function of CD4 T-cells during *T. cruzi* infection

**Figure 2:**
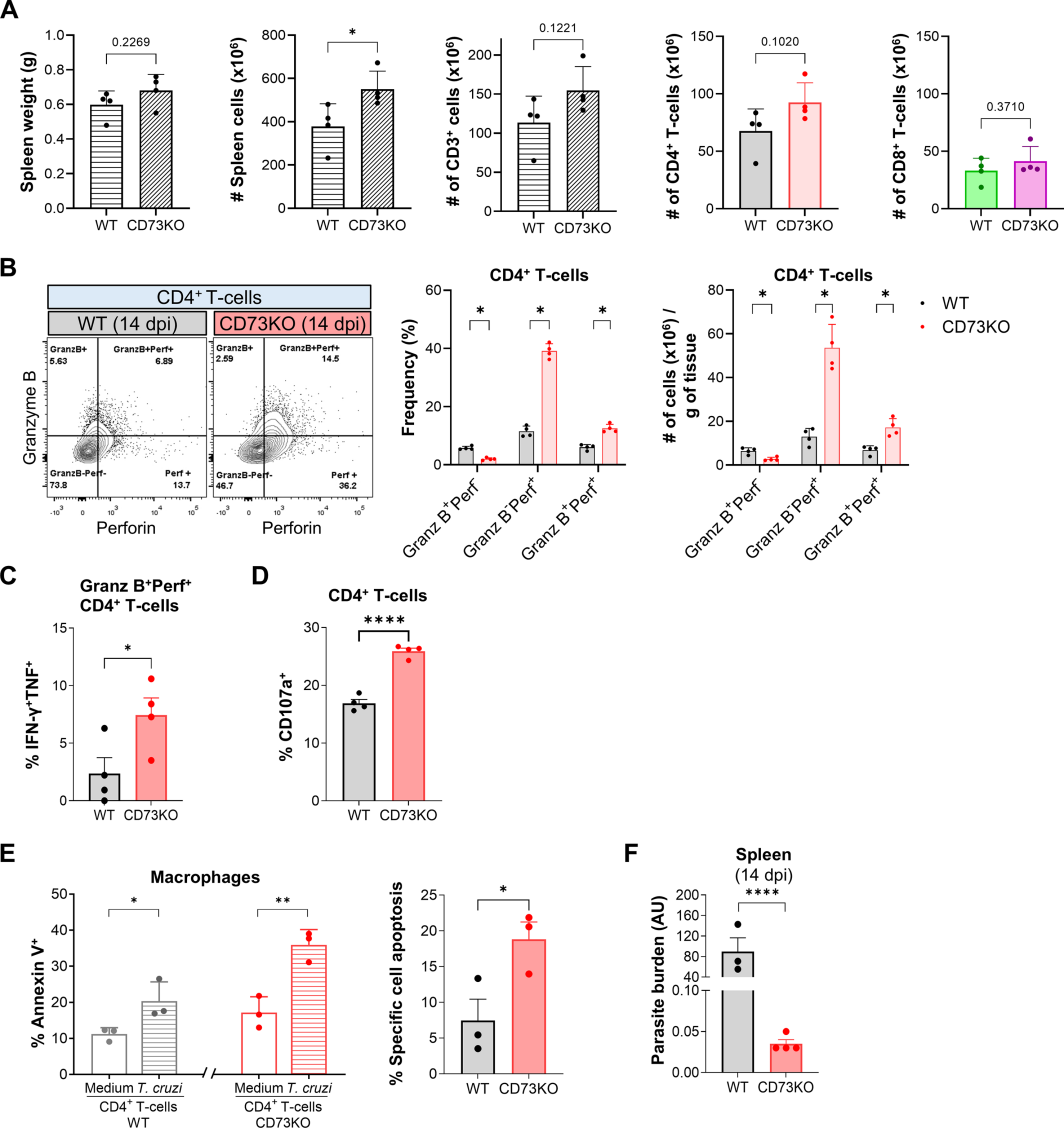
CD73 deficiency enhances cytotoxic CD4 T-cell response and reduces parasite burden. **(A)** Spleen weight and total numbers of splenic cells, T-cells, CD4^+^ and CD8^+^ T-cells from WT and CD73KO mice at 14 dpi (n = 4). **(B)** Representative contour plots, frequencies and number of cells per gram of tissue of GranzB^+^Perf^-^, GranzB^-^Perf^+^, and GranzB^+^Perf^+^ in CD4^+^ T-cells in spleens from WT and CD73KO mice at 14 dpi. **(C)** Frequencies of IFN-γ^+^TNF-α^+^ in GranzB^+^Perf^+^CD4^+^ T-cells in spleens from WT and CD73KO mice at 14 dpi. **(D)** Frequencies of CD107a^+^ in CD4^+^ T-cells in spleens from WT and CD73KO mice at 14 dpi (n = 4) after *T. cruzi* lysate stimulation. **(E)** CD4^+^ T-cells isolated from the spleens of WT and CD73KO mice at 14 dpi were co-cultured for 4 h with peritoneal macrophages labeled with eFluor-670 (eFluor-670^+^) and infected *in vitro* with *T. cruzi* (“*T. cruzi*”) or maintained in medium (“Medium”) (triplicate per condition). The frequency of apoptotic macrophages (Annexin V^+^) was determined by flow cytometry. The rate of specific cell apoptosis (% Specific cell apoptosis) was calculated as: (Apoptotic macrophages)*_T. cruzi_* - (Apoptotic macrophages)_Medium_. **(F)** Parasite burden in the spleens of WT and CD73KO mice at 14 dpi. Pooled samples (WT: n = 5 mice; CD73KO: n = 4 mice) were quantified by real-time PCR in technical triplicate (WT) or quadruplicate (CD73KO). Results were normalized to endogenous *18S* gene levels and expressed as arbitrary units (AU). Independent samples t-tests were performed to compare WT vs. CD73KO and Medium vs. *T. cruzi*. *p < 0.05, **p < 0.01, ***p < 0.001, ****p < 0.0001.

Building on our recent report showing that ADO regulates granzyme B expression in CD4 T-cells (6), we next sought to determine how CD73 activity shapes CD4 CTL responses during *in vivo T. cruzi* infection. To address this, we performed a comparative analysis of the immune response elicited by the infection in CD73-deficient mice (*Cd73^-/-^*, hereafter CD73KO) and their *Cd73^+/+^* (WT) counterparts. As was observed in WT mice, infection induced a marked increase in spleen cell counts in CD73KO mice, accompanied by an expansion of CD4^+^ T-cells but not CD8^+^ T-cells (**Supplementary Figure 1A**). Furthermore, the frequencies and counts per grams of tissue of CD4 and CD8 T-cells displaying a cytotoxic phenotype significantly increased (**Supplementary Figure 1B**). Notably, the absolute numbers of total CD3^+^, CD4^+^, and CD8^+^ T-cells were comparable between CD73KO and WT groups (**Figure 2A**). However, CD73KO mice displayed a marked increase in both the frequencies and counts per grams of tissue of Perf^+^ and GranzB^+^Perf^+^ CD4 T-cells compared with WT mice (**Figure 2B**). In parallel, the CD4 T-cell compartment of CD73KO mice exhibited a higher multifunctional profile, with an increased proportion of IFN-γ^+^TNF-α^+^ CD4 CTL (**Figure 2C**), and increased CD107a expression following re-stimulation with *T. cruzi* lysate (**Figure 2D**), consistent with an enhanced cytotoxic effector program. This augmented cytotoxic signature was further supported by the overexpression of several markers typically associated with the CD4 CTL phenotype, including the transcription factors T-bet and Eomes, as well as the activation and effector differentiation markers CD39 and CD38 (**Supplementary Figure 1C**). In contrast, the frequency of CD8 T-cells expressing cytotoxic molecules did not differ significantly between WT and CD73KO mice (**Supplementary Figure 1D**). Notably, the degranulation marker CD107a increased in CD8 T-cells after re-stimulation with parasite lysate (**Supplementary Figure 1E**). These findings indicate that CD73-mediated regulation predominantly affects the frequency of CD4 CTLs and the production of cytotoxic effector molecules.

To determine the factors responsible for the divergent responses between the two cytotoxic populations, we examined the expression of the ATP receptor P2X7 on splenic T-cells from infected mice. The t-SNE analysis revealed that CD4 T-cells segregated into clusters with higher P2X7 expression than CD8 T-cells (**Supplementary Figure 2A**). To further quantify this observation, we performed a complementary analysis of P2X7 expression across defined T-cell subsets. CD4^+^ T-cells represented the major population (~60% of total T-cells), with approximately 53% of these cells expressing P2X7. In contrast, only ~13% of CD8^+^ T-cells were P2X7^+^, while CD4^-^CD8^-^ T-cells accounted for a minor fraction (~3%). Additionally, P2X7 expression levels were significantly higher on CD4 than on CD8 T-cells. These results indicate that, although P2X7R expression is present across multiple T-cell subsets, it is both more frequent and more abundant within the CD4 T-cell compartment, supporting a preferential association of P2X7R signaling with CD4 T-cell responses during infection.

Given that ADO regulates the T-cell functions (21), we next examined whether the enhanced cytotoxic profile of CD73-deficient CD4 T-cells translated into improved control of *T. cruzi*-infected targets. To this aim, splenic CD4 T-cells isolated from infected WT or CD73KO mice were co-cultured with *in vitro*-infected or uninfected peritoneal macrophages. After 4 h of incubation, a higher proportion of apoptotic macrophages was detected in cultures with infected macrophages compared with uninfected macrophages. Notably, CD73KO CD4 T-cells induced a significantly higher apoptotic rate than WT CD4 T-cells (**Figure 2E**). We further validated these results by measuring lactate dehydrogenase (LDH) released into culture supernatants as an indicator of cell lysis. Cultures with CD73KO CD4 T-cells exhibited higher LDH levels than those with WT cells (**Supplementary Figure 2B**), reinforcing the concept of an enhanced cytotoxic potential of CD73-deficient CD4 T-cells. Consistent with these *in vitro* findings, CD73KO mice displayed a marked reduction in splenic parasite burden at 14 dpi compared with WT (**Figure 2F**).

These findings suggest that CD73 activity regulates the differentiation and effector programming of CD4 CTLs, resulting in reduced cytotoxic potential and the concomitant parasite persistence during acute infection.

### 2.3 CD73 deficiency increases the frequency of CD4 CTL into cardiac tissue during *T. cruzi* infection

**Figure 3:**
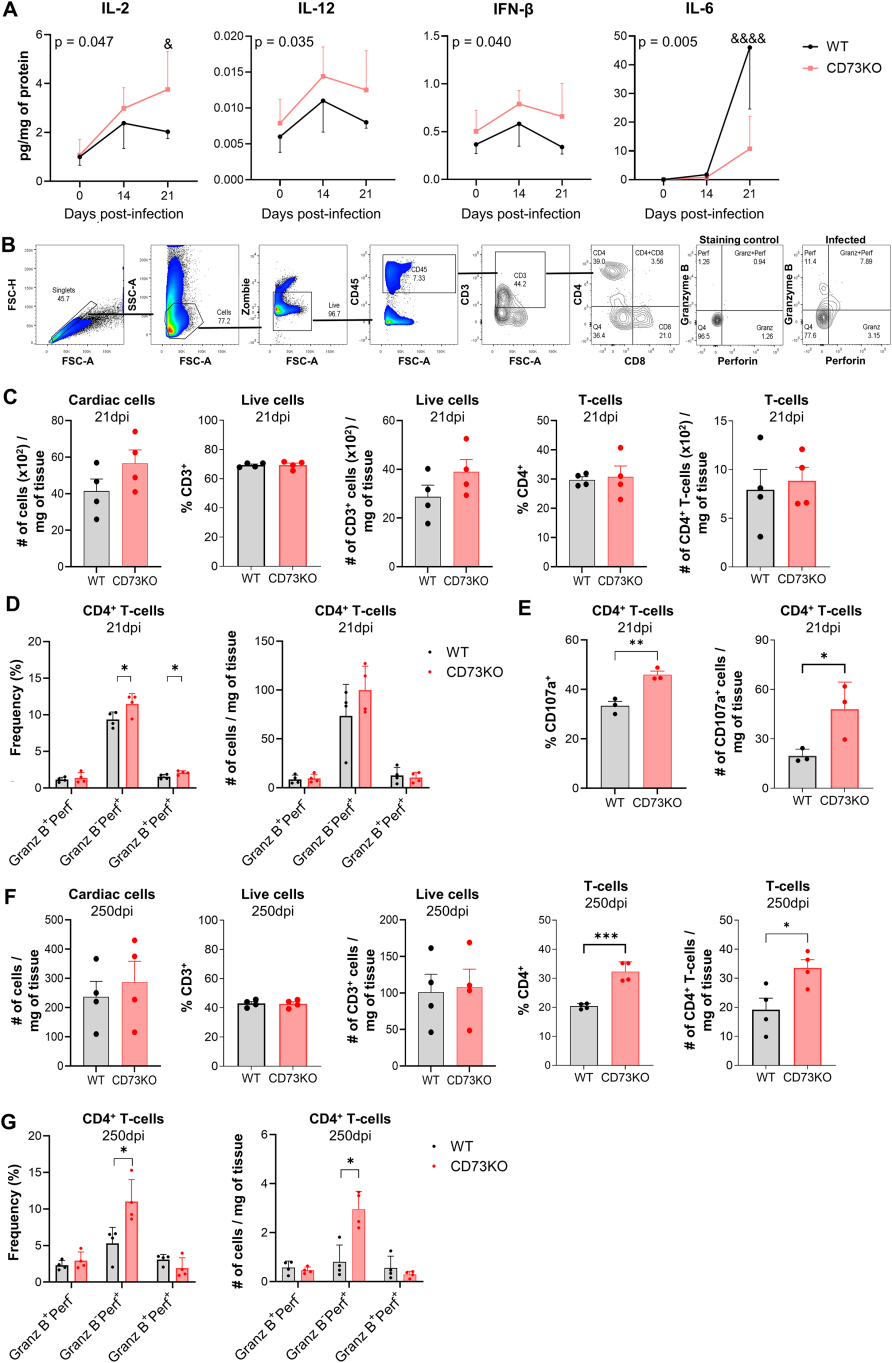
CD73 deficiency drives CD4 CTL accumulation in infected cardiac tissue. **(A)** IL-2, IL-12, IFN-β, and IL-6 levels in cardiac tissue from WT and CD73KO mice at 0, 14 and 21 dpi, normalized to total protein concentration (n = 4 per time point per group). **(B)** Representative gating strategy used to identify cardiac T-cells with cytotoxic phenotype by flow cytometry. Comparisons between infiltrating cells in cardiac tissue from WT and CD73KO mice at 21 dpi **(C–E)** (n = 3–4 per group) and at 250 dpi **(F, G)** (n = 4 per group). **(C–F)** Number of infiltrating cells per milligram of tissue, and frequencies and numbers of T-cells and CD4^+^ T-cells per milligram of tissue. **(D–G)** Frequencies and numbers of GranzB^+^Perf^-^, GranzB^-^Perf^+^, and GranzB^+^Perf^+^ in CD4^+^ T-cells per milligram of tissue. **(E)** Frequency and number of CD107a^+^ cells in CD4^+^ T-cells per milligram of tissue. A: Data were analyzed using a two-way ANOVA followed by Šídák’s *post hoc* multiple comparisons test. The p-values shown in each figure correspond to the main effect of condition (WT vs. CD73KO). Symbols & and &&&& indicate significant differences from CD73KO at the specified time points (p < 0.05 and p < 0.0001, respectively). C-G: Independent samples t-tests were performed to compare WT vs. CD73KO. *p < 0.05, **p < 0.01, ***p < 0.001.

Previous studies have shown that CD4 CTLs preferentially accumulate in tissues with high antigen loads, where they contribute to local infection control (22, 23). Based on this, we hypothesized that *T. cruzi* infection generates a cardiac microenvironment that favors CD4 CTL infiltration and expansion. Indeed, cardiac tissue from CD73KO mice displayed a more pronounced inflammatory milieu than their WT counterparts, characterized by increased levels of IL-2, IL-12, and IFN-β, together with reduced IL-6 (**Figure 3A**). Moreover, we previously reported elevated IFN-γ levels in CD73KO cardiac tissue (15). This cytokine profile has been associated with CD4 CTL differentiation (24–26).

Using the gating strategy shown in **Figure 3B**, we observed no differences between the proportion of CD4 T-cells (**Figure 3C**), however, CD73KO mice exhibited higher cardiac frequencies of Perf^+^ and GranzB^+^Perf^+^ CD4 T-cells during the acute phase of infection (21 dpi) compared with WT mice (**Figure 3D**). In addition, more than 30% of CD4 T-cells in both strains expressed the degranulation marker CD107a (**Figure 3E**), indicating a functionally active cytotoxic phenotype. Notably, CD73KO mice showed a significantly greater number of CD107a^+^ CD4 T-cells than WT counterparts, suggesting that the abrogation of CD73 activity further amplifies their cytotoxic potential. In contrast, no significant differences were found in the cytotoxic potential of KO and WT CD8 T-cells (**Supplementary Figures 2A, B**), suggesting that also in the cardiac tissue, CD73 activity selectively affects the CD4 T-cell compartment.

Next, we evaluated cytotoxic T-cell populations during the chronic stage of cardiac infection. At 250 dpi, CD73KO hearts exhibited a higher proportion of CD4 T-cells (**Figure 3F**), along with a relative reduction in CD8 T-cells (**Supplementary Figure 2C**). In line with this, the Perf^+^ CD4 T-cell subset remained significantly enriched in CD73KO hearts (**Figure 3G**), with no differences in cytotoxic CD8 T-cell between groups (**Supplementary Figure 2D**).

These findings suggest that CD73 acts as a key regulator of CD4 CTL functional capacity during *T. cruzi* infection, fostering their presence in cardiac tissue and their persistence during chronic disease stages.

### 2.4 CD73 deficiency enhances the cardiac anti-parasite response but is associated with altered cardiac function

**Figure 4:**
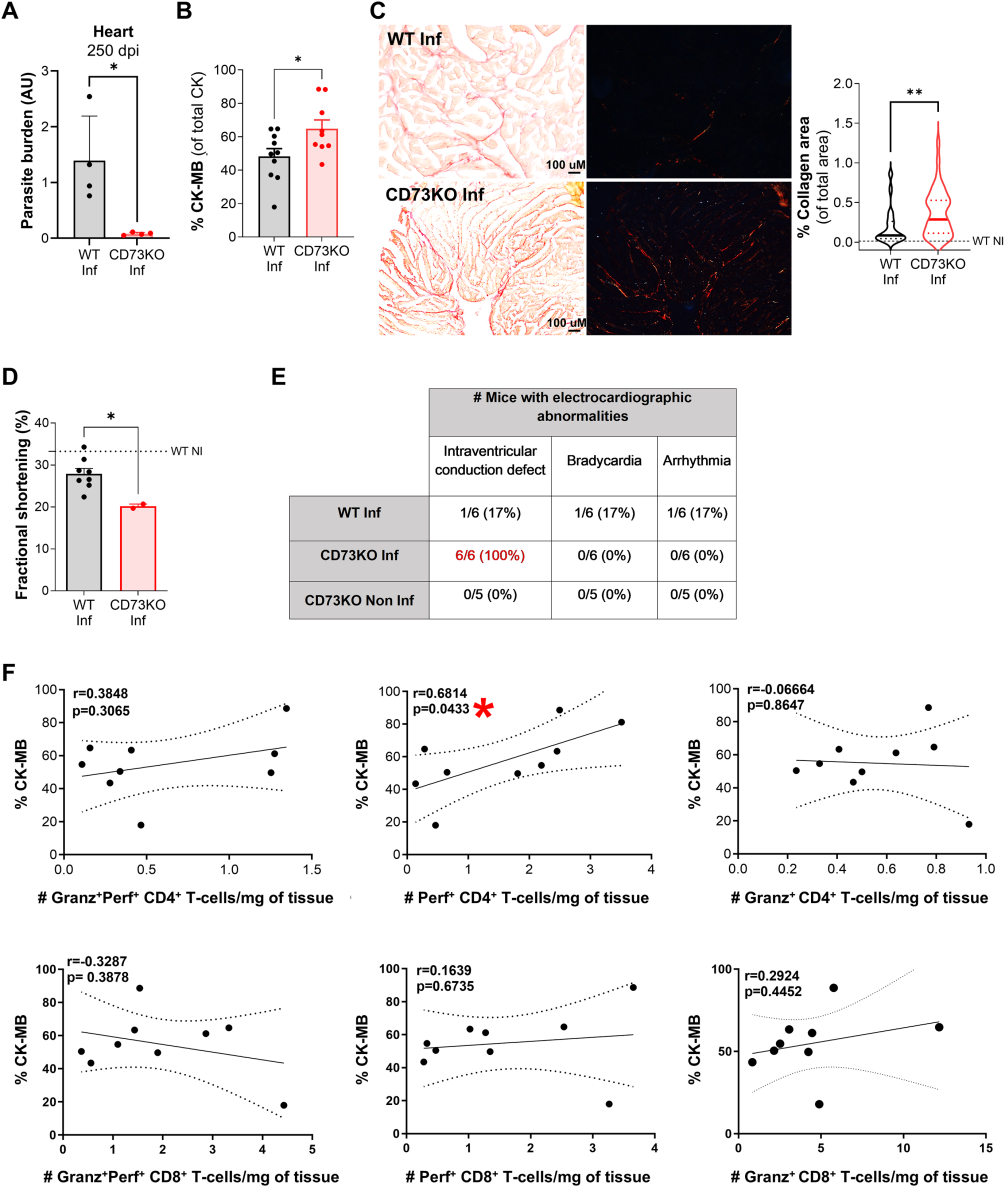
CD73 deficiency is associated with enhanced parasite control but exacerbated cardiac injury. **(A)** Cardiac parasite burden in WT and CD73KO mice at 250 dpi. Pooled samples (WT: n = 3; CD73KO: n = 4) were quantified by real-time PCR in technical quadruplicates. Results were normalized to endogenous *18S* gene levels and expressed as arbitrary units (AU). **(B)** Plasma levels of the CK-MB isoform relative to total CK in WT and CD73KO mice at 250 dpi (n = 9–10 per group). **(C)** Representative images (10x) under bright-field and polarized light illustrate collagen deposits at 250 dpi stained with picrosirius red. Quantification of myocardial collagen deposition in cardiac tissue from WT and CD73KO mice. Measurement of uninfected age-matched WT mice (“WT NI”) is shown with a dashed line. **(D)** Left ventricular contractile function assessed as fractional shortening in WT (n = 8) and CD73KO (n = 2) mice at 250 dpi. Measurement of uninfected age-matched WT mice is shown with a dashed line (“WT NI”) (n = 8). **(E)** Number of mice with electrocardiographic abnormalities in WT and CD73KO groups at 250 dpi (n = 6 per group) and uninfected age-matched CD73KO controls (n = 5). **(F)** Correlations between plasma CK-MB levels and the numbers of GranzB^+^Perf^+^, GranzB^-^Perf^+^, and GranzB^+^Perf^-^ CD4^+^ (top panel) or CD8^+^ (below panel) T-cells in cardiac tissue from WT and CD73KO mice (n = 9) at 250 dpi. A-C: Independent samples t-test was performed to compare WT vs. CD73KO (*p < 0.05, **p < 0.01). D: Mann-Whitney test was performed to compare WT and CD73KO infected mice. F: Pearson’s test was performed to analyze correlations. The solid line indicates the correlation trend, while dashed lines represent the 95% confidence interval.

Consistent with the increased presence of CD4 CTLs in cardiac tissue during both acute and chronic phases of infection, CD73KO mice exhibited a marked reduction in cardiac parasite burden at 250 dpi (**Figure 4A**). However, relative plasma levels of creatine kinase MB (CK-MB), a sensitive biomarker associated with myocardial injury, were significantly higher in CD73KO mice than in WT (**Figure 4B**).

Myocardial fibrosis, a key histopathological hallmark of Chagas cardiomyopathy, is strongly correlated with impaired cardiac function and adverse clinical outcomes (27). To gain mechanistic insight into this phenotype, we evaluated components of the Wnt signaling pathway in cardiac tissue and found that infected CD73KO hearts exhibited significantly higher transcript levels of *Wnt3a* and *Wnt5a* than those in infected WT mice (**Supplementary Figure 4A**). The results support the activation of profibrotic Wnt signaling pathways known to promote fibroblast activation and extracellular matrix deposition (28). In line with these molecular changes, cardiac tissue from CD73-deficient mice displayed increased collagen deposition, indicating enhanced fibrotic remodeling (**Figure 4C**).

In agreement with these structural alterations, preliminary echocardiographic analyses revealed reduced contractile capacity in infected mice, as evidenced by decreased fractional shortening compared with uninfected WT mice. Notably, data obtained from infected CD73KO mice suggest a more pronounced functional impairment than infected WT controls (**Figure 4D**).

Electrocardiographic assessments further demonstrated a higher frequency of conduction abnormalities in infected CD73KO mice compared with both uninfected CD73KO and infected WT groups (**Figure 4E**). Although heart rate and PR and QT intervals remained comparable among infected mice, all infected CD73KO mice displayed alterations in the QRS complex (**Supplementary Figures 4B, C**), including reduced amplitude suggestive of intraventricular conduction defects. Importantly, none of these abnormalities were observed in age-matched uninfected CD73KO mice, indicating that cardiac dysfunction is directly associated with infection and likely driven by the exacerbated inflammatory response observed in the absence of CD73 activity.

Given previous evidence linking CD4 CTLs to myocardial injury in Chagas disease (7, 29), we next explore the potential contribution of cytotoxic T-cell infiltration to tissue damage. As shown in **Figure 4F**, the frequency of Perf^+^ CD4 T-cell positively correlated with plasma CK-MB levels, whereas no significant associations were detected for cytotoxic CD8 T-cells. Although the underlying mechanisms remain to be fully clarified, these findings suggest that CD4 CTLs, while contributing to parasite control, may also promote tissue injury during the chronic stage of infection.

### 2.5 Chronic Chagas disease patients display a higher percentage of circulating granzyme B^+^ CD4 T-cells

**Figure 5:**
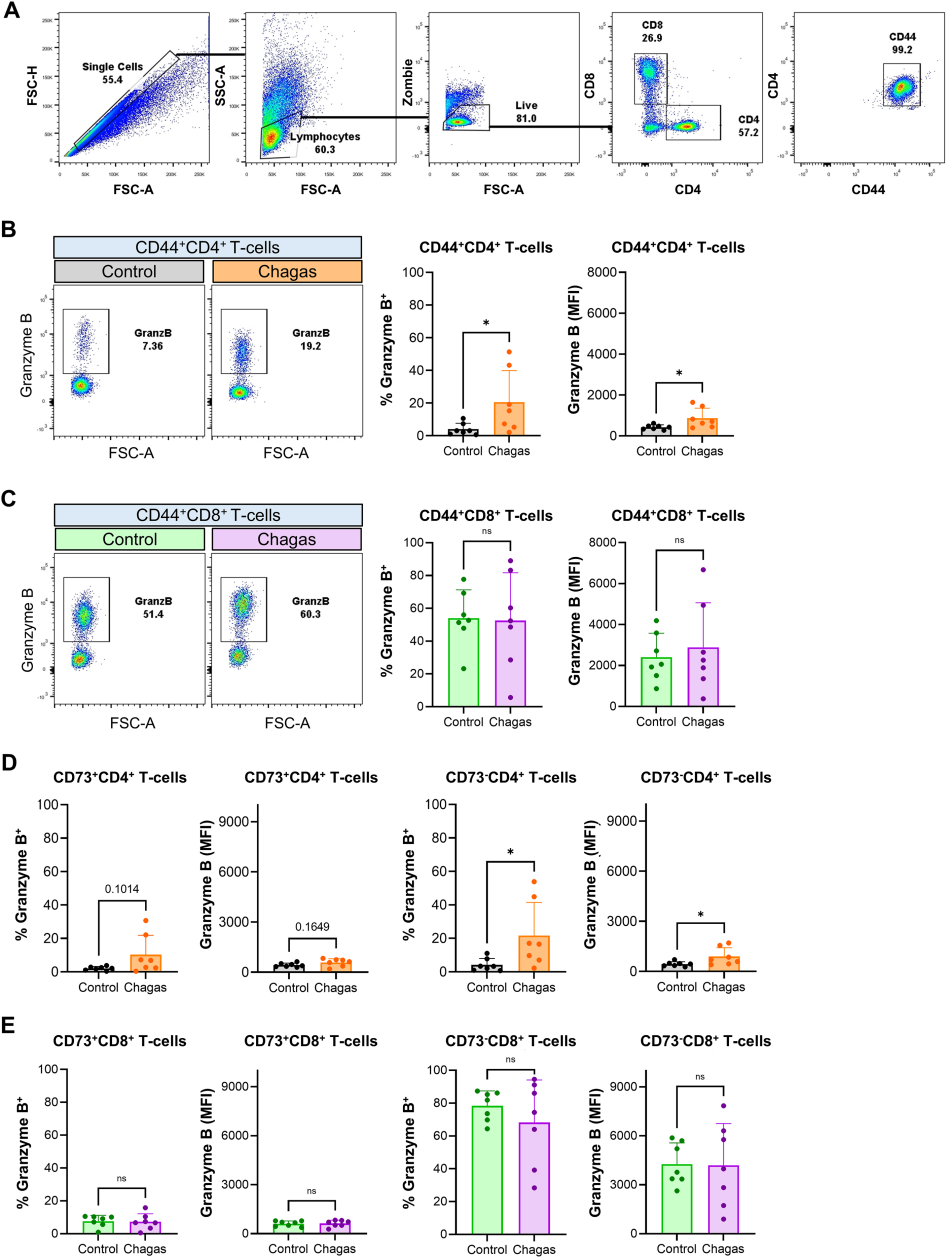
Patients with chronic Chagas disease display an increased frequency of circulating granzyme B^+^ CD4^+^ T-cells. **(A)** Gating strategy for the analysis of the PBMCs isolated from 7 adult patients with asymptomatic chronic Chagas disease (“Chagas” group, 5 males and 2 females) and 7 adult seronegative individuals (“Control” group, 3 males and 4 females) stimulated with anti-CD3/CD28 for 72 h. **(B, C)** Representative dot plots and frequencies of granzyme B^+^ cells in CD44^+^CD4^+^ **(B)** and CD44^+^CD8^+^ **(C)** T-cells and granzyme B expression levels (MFI) by flow cytometry. **(D, E)** Frequencies of granzyme B^+^ cells or granzyme B expression levels (MFI) in CD4^+^ **(D)** or CD8^+^ **(E)** T-cells expressing CD73 (CD73^+^) or their counterpart CD73^-^. Mann-Whitney test was performed to compare Control vs. Chagas. *p < 0.05.

In order to study the CD4 T-cells with cytotoxic potential in infected patients, we analyzed PBMCs from patients with chronic *T. cruzi* infection. Because granzyme B expression is low or absent in resting circulating human CD4^+^ T-cells (30), PBMCs isolated from *T. cruzi*-seropositive individuals without clinical manifestations (“Chagas” group) and from seronegative donors (“Control” group) were stimulated with anti-CD3/CD28 antibodies. After 72h, activated (CD44^+^) T-cells were characterized following the gate strategy shown in **Figure 5A**.

Both CD4 and CD8 T-cells from the two groups responded to TCR stimulation. However, CD4 T-cells from the Chagas group exhibited significantly higher expression of the activation marker CD44 compared with the Control group, a difference not observed in CD8 T-cells (**Supplementary Figure 5A**). Notably, CD4 T-cells from the Chagas group displayed increased granzyme B expression relative to the Control group, whereas no differences were detected in the CD8 compartment (**Figures 5B, C**).

Analysis of purinergic components revealed a decreased frequency of CD39^+^CD73^-^ CD4 T-cells in the Chagas group compared with controls, while CD73 expression and the frequency of CD39^+^CD73^+^ double-positive cells remained unchanged (**Supplementary Figure 5B**). These findings align with our previous report showing elevated plasma ATP levels in chronic Chagas patients (31) and suggest reduced capacity of CD4 T-cells from infected individuals to metabolize extracellular ATP.

Stratifying of CD4 T-cells by CD73 expression we found that the CD73^-^ subset displayed a markedly higher proportion of granzyme B^+^ cells in the Chagas group compared with the Control group (**Figure 5D**). In contrast, CD8 T-cells showed no differences between groups in purinergic component expression or in granzyme B levels (**Figure 5E**; **Supplementary Figure 5C**). This pattern parallels our observations in the murine model and supports an association between reduced CD73 expression and enhanced cytotoxic potential in CD4 T-cells during chronic infection.

These findings indicate that patients with chronic asymptomatic infection display an enhanced cytotoxic potential among circulating CD4 T-cells, particularly within the CD73^-^ subset. These results support a systemic role for purinergic signaling in regulating CD4 CTL differentiation and function in human chronic infection.

### 2.6 Cardiac tissue from patients with end-stage CCC shows enrichment of cytotoxic response gene expression

Despite advances in understanding the pathogenic mechanisms underlying CCC, these processes remain incompletely defined. It has been proposed that disease progression results from insufficient parasite clearance, leading to persistent inflammation and progressive myocardial injury (32). Previously, we demonstrated that purinergic immunoregulatory pathways are activated in the cardiac tissue of CCC patients (6). Given our current findings showing that CD4 CTL accumulation correlates with tissue damage, we hypothesized that CD4 CTLs may contribute to pathogenic mechanisms in human CCC.

To investigate this possibility, we performed a transcriptomic analysis using a public RNA-seq dataset reported by Brochet et al. (33). This dataset included eight myocardial tissue samples from adult patients with end-stage CCC (“CCC” group; five females and three males) and six samples from healthy adult donors (“CTRL” group; six males). Differentially expressed genes (DEGs) between CCC and CTRL groups were identified using DESeq2. No sex-related differences were observed among the DEGs within the CCC group, as was previously described (6).

**Figure 6:**
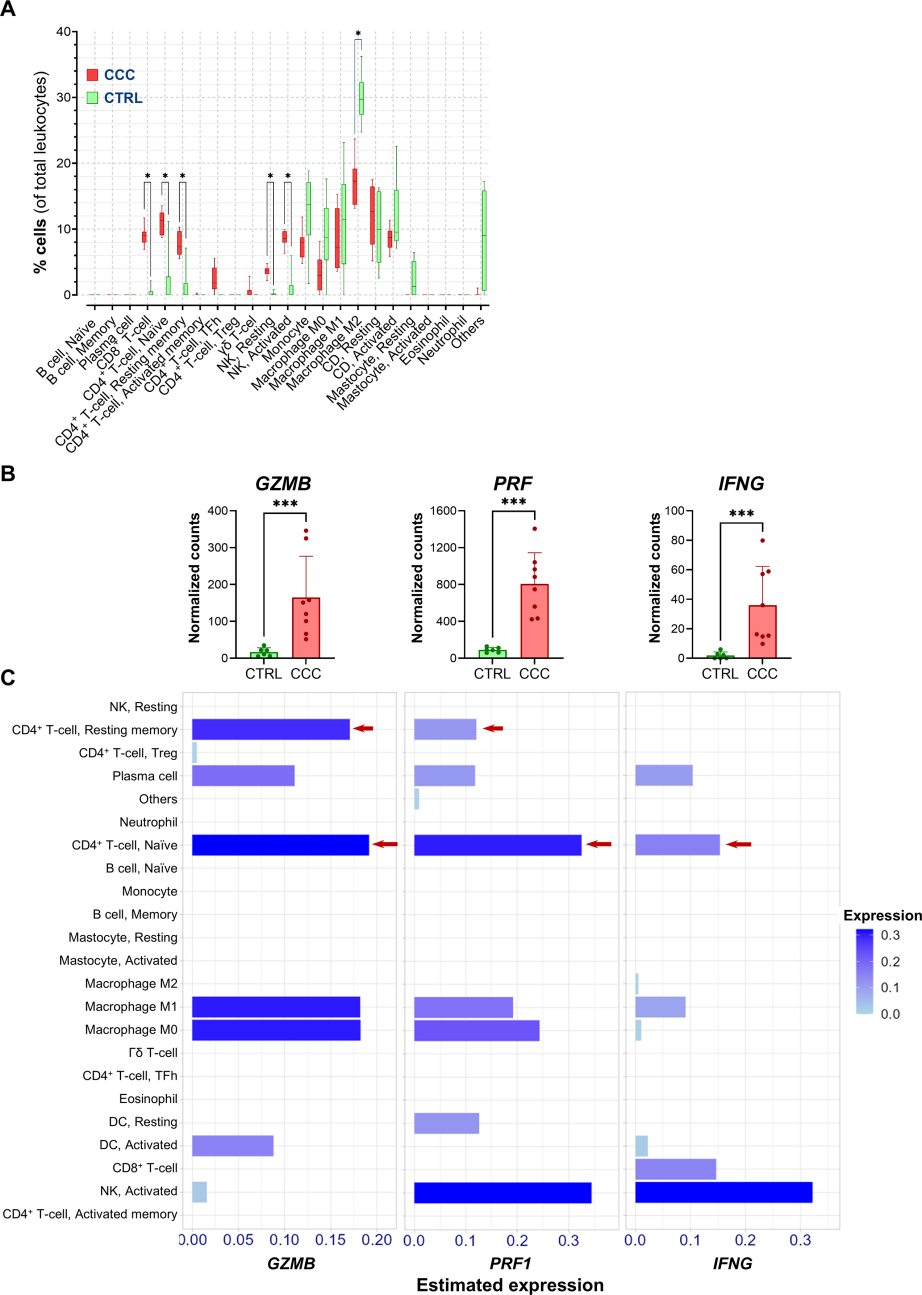
Enrichment of the cytotoxic response in cardiac tissue from CCC patients. RNA-seq data were analyzed from the publicly available dataset GSE191081, which contains myocardial tissue samples from patients with end-stage Chagas cardiomyopathy (“CCC” group, n = 8) and control donors (“CTRL” group, n = 6). **(A)** Estimation of leukocyte proportions by cell deconvolution using the LM22 transcriptional signature matrix. **(B)** Transcript levels of *GZMB*, *PRF1*, and *IFNG* in cardiac tissue from each group. **(C)** Enrichment of *GZMB*, *PRF1*, and *IFNG* transcripts across different leukocyte populations inferred from the deconvolution analysis in CCC group. Mann-Whitney test was performed to compare Control vs. Chagas. *p < 0.05, ***p < 0.001.

To characterize the immune infiltrate, we applied the LM22 leukocyte signature matrix for cell-type deconvolution. CCC samples displayed increased proportions of naïve and memory CD4 T-cells, CD8 T-cells, and NK cells, accompanied by a reduction in M2 immunosuppressive macrophages, suggesting a predominantly inflammatory and cytotoxic immune microenvironment in the myocardium (**Figure 6A**).

To further define the cellular sources of CD73 within infiltrating cells, we analyzed *NT5E* transcript distribution across the deconvoluted immune populations (**Supplementary Figure 6**). This analysis showed that the highest estimated *NT5E* expression on a per-cell basis was observed in resting NK cells and CD4 T-cells. However, the NK population represents only a minor fraction of the total CD45^+^ infiltrate in CCC myocardial tissue (3.62%; **Figure 6A**). In contrast, naïve CD4 T-cells, which also exhibit substantial *NT5E* expression, account for a considerably larger proportion of the infiltrating immune population (~11%). Therefore, when both per-cell expression and cellular abundance are considered, CD4 T-cells likely represent a major contributor to overall CD73 transcript levels within the CCC myocardium.

We then examined the expression of transcripts encoding the key effector molecules granzyme B (*GZMB*), perforin (*PRF1*), and IFN-γ (*IFNG*). CCC patients exhibited higher levels of *GZMB*, *PRF1*, and *IFNG* compared with controls, consistent with transcriptional activation of cytotoxic responses (**Figure 6B**). Mapping these transcripts to specific leukocyte subsets revealed CD4 T-cells as a major source of *GZMB*, *PRF1*, and *IFNG*, strongly supporting their active role in end-stage CCC (**Figure 6C**). Additional immune populations, including plasma cells, macrophages, dendritic cells, and NK cells, also expressed cytotoxic transcripts. Notably, no enrichment of *GZMB* or *PRF1* transcripts was detected in CD8 T-cells from CCC patients, suggesting that their cytotoxic program may be impaired at this disease stage, a feature consistent with previous reports of CD8 T-cell exhaustion in CCC (34). These findings were further validated using the “3kPBMC” reference matrix (data not shown).

Collectively, these results demonstrate that cardiac tissue from CCC patients exhibits a transcriptional profile characterized by chronic inflammation and abundant cytotoxic infiltrates, predominantly composed of CD4 rather than CD8 T-cells. Together with the murine findings, these data support the hypothesis that dysregulated purinergic signaling and CD4 CTL accumulation may contribute to chronic myocardial damage in advanced disease stages.

## 3 Discussion

In the present study, we comprehensively investigated the role of CD73 in shaping the development, effector functions, and pathogenic potential of cytotoxic CD4 T-cells during *T. cruzi* infection. Our findings suggest that CD4 CTLs play a dual and context-dependent role: they contribute to parasite control, but they can also mediate tissue injury and cardiac pathology during chronic stages. Understanding the signals that regulate this balance is therefore critical for elucidating the immunobiology of infectious cardiomyopathy and identifying rational targets for immunomodulation.

Our group has extensively examined the interplay between *T. cruzi* infection and purinergic signaling in both experimental models and patients with Chagas disease (6, 14, 20, 31, 35, 36). Persistent exposure of parasite antigens, together with dysregulated inflammation are recognized drivers of tissue damage in CCC (32). Given the central role of extracellular ATP and ADO as metabolic cues that coordinate inflammatory versus regulatory pathways, we hypothesized that CD73, which generates ADO, acts as a molecular checkpoint that modulates cytotoxic T-cell responses. Consistent with this concept, our previous work established that ATP-P2X7 signaling promotes CD4 T-cell effector differentiation, while ADO-A2aR signaling exerts an opposing immunoregulatory role (6). Moreover, we showed that CD73 expression within cardiac tissue is confined to immune cells, with T-cells representing the principal immune population during *T. cruzi* infection (35). Building on these observations, we now demonstrate that CD73 actively restrains the *in vivo* differentiation and cytotoxic programming of CD4 T-cells, potentially impacting both parasite clearance and cardiac immunopathology.

During acute infection, we previously demonstrated that the CD73^-^ CD4^+^ T-cell compartment exhibits a higher frequency of granzyme B-producing cells than its CD73^+^ counterparts (6). In the present study, we further demonstrated that CD73 deficiency led to a marked expansion of CD4 CTLs accompanied by an enhanced multifunctional cytotoxic profile. Importantly, this expansion was highly selective for the CD4 compartment, as the frequency of cytotoxic CD8 T-cells remained unchanged. These observations reinforce the concept that CD4 T-cells are particularly sensitive to purinergic signals, likely due to their higher expression of the purinergic receptors P2X7 and A2a (37, 38). In line with this notion, our data show that P2X7R expression is predominantly segregated within the CD4 T-cell population, a receptor previously associated with T-cell activation and proliferative capacity (6). Accordingly, CD73-generated ADO emerges as a key negative regulator of CD4 CTL differentiation in the context of myocardial infection, a mechanism first described by our group (6). Although several aspects of CD4 T-cells were targets of CD73 activity, we found that the frequency of splenic CD8 T-cells expressing CD107 also increases in CD73KO mice compared with WT mice, suggesting that these cells may contribute to controlling the infection in the KO mice. Given the growing interest in targeting CD73 in oncology and chronic inflammatory diseases (39, 40), our findings highlight the relevance of this pathway in parasite infections (or in cardiac tissue) and raise translational considerations.

The dual nature of CD4 CTLs has been increasingly recognized. These cells contribute to pathogen control but can also promote immunopathology in chronic infection, autoimmunity, and cardiac inflammatory disorders (7, 41, 42). In Chagas disease, it is well established that immune events during the acute phase strongly influence the progression toward chronic cardiomyopathy (8, 43). In the present work, we observed that CD4 CTLs persist in the heart during chronic *T. cruzi* infection, and their frequency correlates with circulating tissue damage markers such as CK-MB. While further experiments are necessary to conclude that sustained CD4 CTL activity drives progressive myocardial injury during *T. cruzi* infection, similar phenomena have been described in other chronic infections, in which prolonged antigen exposure maintains cytotoxic programs that ultimately damage host tissue (7–9, 29).

This pathogenic dimension was particularly evident in CD73KO mice, which, despite improved parasite control, exhibited exacerbated myocardial fibrosis, reduced contractility, and more pronounced electrocardiographic alterations. In accord with our findings, global CD73 deficiency in models of sterile myocarditis and ischemia/reperfusion impairs tissue protection and repair (44, 45). Moreover, Quast et al. demonstrated that ADO generated by CD73 specifically on T-cells, limits the production of inflammatory cytokines and fibroblast activation within the myocardium, thereby preventing pathological remodeling. Notably, global and T-cell-specific CD73^-/-^ mice displayed comparable functional impairment following cardiac injury, strongly suggesting that CD73 on T-cells plays a major role during tissue remodeling (46). Together, our data identify CD73/ADO signaling as a central immunometabolic axis that orchestrates the balance between protective cytotoxic immunity and cardiac homeostasis. In its absence, enhanced CD4 CTL activity may control parasite burden -although the involvement of other immune mechanisms cannot be excluded- at the cost of aggravating chronic cardiac pathology.

In human disease, we provide complementary evidence supporting the murine findings. Patients with chronic Chagas disease exhibited increased granzyme B expression in circulating CD4 T-cells, particularly within the CD73^-^ subset, whereas no significant differences were observed in CD8^+^ T-cells compared with controls. Elevated plasma ATP levels have been previously reported in Chagas patients (31), further support a pro-inflammatory purinergic environment that could favor CD4 CTL differentiation. The presence of a cytotoxic CD4 program, even in asymptomatic individuals, may suggest a protective mechanism that limits parasite spread but also raises the possibility that this response predisposes to immune-mediated myocardial injury.

At the tissue level, the RNA-seq deconvolution analysis of myocardium from end-stage CCC patients revealed marked enrichment of cytotoxic transcripts within the cardiac infiltrate, with naïve and memory CD4 T-cells emerging as the principal cellular sources, whereas CD8 T-cells mimicked an exhausted phenotype with no enrichment of these effector molecules. In parallel, analysis of CD73 transcript (*NT5E)* suggested that the overall contribution to CD73 levels is particularly driven by naïve CD4 T-cells. In concordance with our prior histological evidence showing that CD73 expression localizes predominantly in infiltrating immune cells rather than resident cardiac cells (35), these findings support a model in which immune cell-derived CD73 is a key regulator of the myocardial microenvironment in CCC.

Notably, ADO signaling has been implicated in promoting fibroblast activation and extracellular matrix deposition (46–48), providing a mechanistic link between purinergic metabolism, cytotoxic infiltration, and chronic tissue remodeling. In line with several studies associating higher *T. cruzi* parasite burden with increased myocarditis severity (20, 49–52), our findings reinforce the concept that CCC myocardium is characterized by an imbalanced immune response, in which CD4 T-cells play a dual role: as key effectors of cytotoxicity and as major contributors to local purinergic signaling. This dual functionality positions CD4 T-cells at the center of the balance between anti-parasite response and immune-mediated tissue damage.

Overall, our study demonstrates that CD73 activity critically modulates the differentiation and effector function of CD4 CTLs, shaping their protective role during acute infection and their pathogenic potential during chronic stages. This duality underscores a major therapeutic challenge: any intervention targeting purinergic pathways must preserve anti-parasite immunity while preventing excessive tissue damage. As CD4 CTLs are emerging as therapeutic targets in oncology and chronic inflammatory diseases (53, 54), our findings provide a conceptual framework for considering CD73 as a metabolic checkpoint. Future therapeutic strategies may benefit from combining purinergic modulators with antiparasitic chemotherapy to simultaneously enhance parasite control, restrain immune-mediated injury, and improve long-term cardiac outcomes.

## 4 Materials and methods

### 4.1 Sex as a biological variable

Sex was considered as a biological variable; however, only female murine samples were available for this study to maintain consistency with previous works (6, 14, 55, 56).

In the experiments using human peripheral blood mononuclear cells (PBMCs) sex was considered as a biological variable in the analyses.

The gene expression dataset (GEO: GSE191081) was obtained from 8 end-stage CCC patients (5 females and 3 males) and 6 male control donors. No sex-dependent differences among the differentially expressed genes (DEGs) within the CCC group were found (6). Therefore, samples from CCC patients were analyzed collectively and compared directly with those from the control group.

### 4.2 Mice

C57BL/6 J (WT) mice were obtained from the Facultad de Ciencias Veterinarias at the Universidad Nacional de La Plata, Argentina. CD73-deficient mice (CD73KO; B6.129S1-Nt5e^tm1Lft^/J - JAX stock #018986) were acquired from The Jackson Laboratories, United State.

All mice were housed in a specific pathogen-free unit on a 12 h light/12 h dark cycle. Environmental conditions were kept constant, with room temperature set at 25 °C and humidity controlled between 40-60%. Throughout the experiments, animals had unrestricted access to food and water. At final time point, mice were anesthetized with isoflurane and then euthanized by cervical dislocation.

### 4.3 Human samples

PBMCs were collected from adult volunteers recruited at Hospital Nuestra Señora de la Misericordia and the Laboratorio Central of Córdoba, Argentina. PBMCs were isolated from venous blood by density gradient centrifugation using Ficoll-Hypaque PLUS (GE Healthcare Bioscience) and frozen at -80 °C. Samples were obtained from 7 individuals with asymptomatic chronic Chagas disease (5 males and 2 females; age 45–65 years, median 50). Infection status was confirmed through a combination of positive indirect hemagglutination and ELISA assays. All infected donors underwent clinical assessments, including electrocardiography (ECG) and chest X-ray, and none presented abnormalities. PBMCs from the control group were collected from 7 seronegative adults (3 males and 4 females; age 28–52 years, median 42). Individuals with chronic or inflammatory conditions, an erythrocyte sedimentation rate >30 mm/h, or leukocyte counts <4, 000 or >10, 000 cells/mm³ were excluded.

Gene expression analysis were performed using the dataset published by Brochet et al. (33). This dataset includes transcriptomic profiles from human left ventricular free wall tissue obtained from patients with end-stage chronic Chagas cardiomyopathy (CCC; 5 females and 3 males) at the time of heart transplantation. Control samples (CTRL; 6 males) were obtained from organ donors with no compatible recipient. Additional information on patient characteristics is available in Brochet et al., 2022. The dataset is accessible under accession number GSE191081 in the Gene Expression Omnibus (GEO).

### 4.4 Experimental infection models

Bloodstream trypomastigotes of *T. cruzi* (Tulahuen strain) were collected from anesthetized infected mice by cardiac puncture and maintained through successive passages. For *in vivo* infection experiments, female mice aged six to eight weeks were intraperitoneally inoculated with 1, 000 blood-derived trypomastigotes. Noninfected mice served as controls.

For coculture assays, blood-derived trypomastigotes were used to infect Vero cell monolayers. After seven days, the supernatants containing trypomastigotes were harvested, washed twice with PBS, and subsequently used to infect macrophages.

### 4.5 Splenocytes and CD4 T-cell isolation

Spleens were aseptically harvested from euthanized mice, collected in cold PBS and mechanically dissociated through a 70 μm nylon mesh to obtain single-cell suspensions. Red blood cells were lysed using a lysis buffer (Gibco). Viable cells were quantified by Trypan blue exclusion using a Neubauer chamber. Total CD4^+^ T-cells were isolated by negative selection from splenic single-cell suspensions using magnetic bead separation (MojoSort, BioLegend).

### 4.6 Cardiac immune cells isolation

Heart leukocyte isolation was performed as previously described (6). In brief, hearts were perfused with cold PBS, weighed, and then mechanically disrupted and enzymatically digested with 0.25% trypsin (Sigma). The digested tissue was gently passed through a 40 μm cell strainer to obtain a single-cell suspension. Mononuclear cells were purified by centrifugation over a 35%/70% Percoll bilayer gradient (GE Healthcare). Viable cells were quantified by Trypan blue exclusion using a Neubauer chamber.

### 4.7 Preparation of *T. cruzi* lysate

Trypomastigotes of *T. cruzi* (Tulahuen strain) were harvested from monolayers of infected Vero cell cultures and washed extensively. Parasites were then subjected to four successive freeze–thaw cycles, after which samples were sonicated. Cell debris was removed by centrifugation at 12, 000 x g, and the resulting supernatant was collected and filtered. Protein concentration was determined using the Bradford assay.

### 4.8 Flow cytometry

Surface staining. Cell suspensions were first incubated with viability dyes. Fluorophore-labeled antibody panels (listed in **Supplementary Table 2**) were then added and incubated for 30 min at 4 °C. After washing with staining buffer (PBS supplemented with 5% FBS), samples were acquired on a BD LSRFortessa flow cytometer, and data were analyzed using FlowJo v10 software (Tree Star, Inc.). Gating strategies are shown in **Figures 1A**, **3B**, **5A**.

Intracellular staining. To assess T-cell functionality, cells were cultured in the presence of monensin (GolgiStop, 0.6 μL/ml; BD Biosciences), brefeldin A (GolgiPlug, 1 μL/mL; BD Biosciences), phorbol 12-myristate 13-acetate (1 mg/mL; Sigma), and ionomycin (1 μg/mL; Sigma), for 4 h. After surface staining, cells were fixed and permeabilized using BD Cytofix/Cytoperm and Perm/Wash (BD Biosciences) or the Foxp3 staining buffer (eBioscience), according to the manufacturer’s instructions. Cells were then incubated with antibodies against granzyme B, perforin, IFN-γ, TNF-α, Eomes, and T-bet. Data acquisition was performed as described above.

For antigen-specific degranulation assays, cells were stimulated with *T. cruzi* lysate (13 µg/mL) for 16 h. During the final 4 h of culture, anti-CD107a, monensin (GolgiStop, 0.6 μL/ml; BD Biosciences), and brefeldin A (GolgiPlug, 1 μL/mL; BD Biosciences) were added. Surface staining and data acquisition were performed as described above.

### 4.9 Macrophage and T-cell co-cultures

Peritoneal cells from uninfected WT or CD73KO mice were obtained using a rapid peritoneal lavage and plated onto adherent culture plates. After a 3-hour incubation, nonadherent cells were removed to retain the peritoneal macrophage population. Macrophages were labeled with the supravital dye eFluor670 and either infected with *T. cruzi* trypomastigotes for 3 hours (“*T. cruzi*”) or kept in culture medium alone (“Medium”). Following two washing steps, the cells were rested for 48 hours. CD4 T-cells isolated from WT or CD73KO mice at 14 dpi were then added to the macrophage cultures at an effector-to-target (E/T) ratio of 5/1 and incubated for 4 hours. After coculture, macrophages were stained with Annexin V, and the percentage of eFluor670^+^Annexin V^+^ cells (“Apoptotic macrophages”) was determined by flow cytometry. Specific macrophage apoptosis was calculated using the formula: % Specific cell apoptosis = (% Apoptotic macrophages)*_T. cruzi_* - (% Apoptotic macrophages)_Medium_.

### 4.10 Cytokine analyses

Cardiac tissue lysates were examined for IL-6, IL-12p70, and IFN-β levels using a bead-based multiplex assay (#740446; LEGENDplex) and flow cytometry (FACS Canto II; BD Biosciences), following the manufacturer’s instructions. Standard curves were generated and analyzed with LEGENDplex software. IL-2 concentrations were measured using the ELISA Max IL-2 kit (BioLegend). Cytokine values were normalized to the total protein content of the cardiac tissue, determined through the Bradford assay (Bio-Rad).

### 4.11 Tissue parasite burden

Genomic DNA was isolated from infected spleen and heart tissues using TRIzol reagent (Sigma-Aldrich), according to the manufacturer’s protocol. Quantification of *T. cruzi* satellite DNA was performed by real-time PCR using a TaqMan gene expression assay (Applied Biosystems). The sequences of primers and probe (Invitrogen) were (all listed in the 5′ to 3′ orientation): *T. cruzi* ASTCGGCTGATCGTTTTCGA (forward), AATTCCTCCAAGCAGCGGATA (reverse), and *T. cruzi* probe CACACACTGGACACCAA, previously reported by Piron et al. (2007) (57). Due to the extremely low parasite load, we pooled cardiac DNA per group to maximize assay sensitivity, and two micrograms of pooled genomic DNA were used as the template. The amount of *T. cruzi* satellite DNA was normalized to 18S rRNA (Endogenous 18S rRNA Control Reagent; Applied Biosystems) and expressed as arbitrary units.

### 4.12 Picrosirius red staining for fibrosis detection

Collagen deposition in cardiac tissue was evaluated using picrosirius red staining. Paraffin-embedded heart sections were first deparaffinized and then rehydrated through a series of graded alcohols. The sections were stained for 1 h at room temperature with a 0.1% (w/v) picrosirius red solution saturated in picric acid. Following staining, slides were rinsed in 0.01 N HCl for 2 min and dehydrated through graded alcohol solutions. Samples were then cleared in two changes of xylene (2 min each) and mounted using Canada balsam. For each tissue, at least 18 images were acquired at 10x magnification using Nikon TE2000U microscope equipped with polarization filters. The collagen content was quantified as the percentage of the total tissue area using ImageJ software (NIH).

### 4.13 Echocardiography and electrocardiography

Echocardiographic (ECHO) assessments were conducted under sedation induced by a single intraperitoneal injection of xylazine (8 mg/kg) and ketamine (90 mg/kg). ECHO recordings were obtained using a LOGIQ e PRO R8 color Doppler ultrasound system (General Electric) equipped with a L8-18i-RS linear transducer (6.7-18.0 MHz). Both M-mode and B-mode images were collected. M-mode tracings were used to determine the end-diastolic diameter (DD) and end-systolic diameter (DS), and fractional shortening (FS) was calculated as FS = [(DD − DS)/DD] x 100.

Electrocardiogram (ECG) assessments were conducted under sedation induced by a single intraperitoneal injection of ketamine (100 mg/kg). Recordings were obtained using a Fukuda Denshi electrocardiograph (Model FD 16) equipped with mouse-adapted electrodes, at a paper speed of 50 mm/s, with the animal placed inside a Faraday cage. The bipolar leads (I, II, and III) and unipolar limb leads (aVR, aVL, and aVF) were recorded. ECG evaluation included measurement of heart rate (bpm), PR and QT intervals, and the identification of intraventricular conduction abnormalities (altered QRS complex amplitude), bradycardia, and arrhythmias.

### 4.14 RT-PCR

RNA was isolated from infected heart tissues using TRIzol reagent (Sigma-Aldrich) and reverse-transcribed into cDNA using a First Strand cDNA Synthesis Kit (#K1651; Thermo Scientific) and GeneAmp PCR System 9700 (Applied Biosystems). Transcripts were quantified by real-time quantitative PCR on a StepOnePlus Real Time PCR System (Applied Biosystems) sequence detector. *Actb* (β-actin) was used as a control gene to calculate the ΔCt values for independent samples. The relative amounts of target/β-actin transcripts were calculated using the 2^−ΔΔCt^ method. These values were then used to calculate the relative expression of specific mRNAs compared with that WT mice. The sequences of primers used are (all listed in the 5′ to 3′ orientation): *Wnt3a* TTCTTACTTGAG GGCGGAGA (forward) and CTGTCGGGTCAAGAGAGGAG (reverse); *Wnt5a* GCA GGA CTT TCT CAA GGA CA (forward) and CCC TGC CAA AGA CAG AAG TA (reverse); *Actb* CGCCAC CAGTTCGCCATGGA (forward) and TACAGCCCGGGGAGCATCGT (reverse).

### 4.15 PBMC activation

PBMCs were thawed and washed twice with PBS, and viable cells were quantified by Trypan blue exclusion using a Neubauer chamber. The cells were then resuspended in RPMI-1640 medium supplemented with 10% FBS, 0.1% gentamicin, and 50 μM β-mercaptoethanol. A total of 4 x 10^5^ cells per well were plated in 96-well plates and stimulated for 72 hours with plate-bound anti-CD3 (2 ug/mL, clone 145-2C11, eBioscience, Cat 16-0031-82) together with soluble anti-CD28 (2 ug/mL, clone CD28.2, eBioscience, Cat 16-0289-85). All cultures were maintained at 37 °C and 5% CO2.

### 4.16 RNA-seq analyses

Statistical analyses, data normalization, and differential gene expression analyses were performed following the methodology previously described (6). Leukocyte population proportions were estimated through cell-type deconvolution of the RNA-seq dataset using the ADAPTS package together with the LM22 reference matrix, which includes transcriptional signatures of 22 mature human hematopoietic cell types.

### 4.17 Statistics

Details on descriptive statistics, including the number of biological or technical replicates and the statistical test applied, are provided within each figure legend. All experiments were conducted at least twice. Sample normality was evaluated using the Shapiro-Wilk test. For comparisons between two independent groups, a two-tailed independent samples t-test was applied, whereas differences among multiple groups were assessed using one-way ANOVA followed by Tukey’s *post hoc* test. Variance homogeneity was examined using the Levene test. Pearson correlation coefficients were used to determine bivariate associations between variables.

A P value below 0.05 was considered statistically significant. Data in the figures are shown as mean ± SD, and significance levels are indicated as: *p < 0.05; **p < 0.005; ***p < 0.001; ****p < 0.0001. All analyses, except for RNA-seq-related processing, were carried out using GraphPad Prism version 9.0 (GraphPad Software).

## Data Availability

Publicly available datasets were analyzed in this study. This data can be found here: GEO database (accession number GSE191081).

## Supplementary material

Supplementary Figure 1 CD73 deficiency no expand the CD8 CTL population **(A)** Spleen weights and total numbers of splenic cells, CD3^+^ cells (T-cells), CD4^+^ and CD8^+^ T-cells from non-infected CD73KO mice (Non Inf, n = 4) and at 14 dpi (Inf, n = 4). **(B)** Representative contour plots, frequencies and number of cells per gram of tissue of GranzB^+^Perf^-^, GranzB^-^Perf^+^, and GranzB^+^Perf^+^ cells in CD4^+^ or CD8^+^ T-cells in non-infected CD73KO mice and at 14 dpi. **(C)** Heatmap comparing the frequencies of CD4^+^ T-cells expressing T-bet, Eomes, CD39, and CD38 between WT and CD73KO mice at 14 dpi (n = 4 per group). **(D)** Representative contour plots, frequencies and number of cells per gram of tissue of GranzB^+^Perf^-^, GranzB^-^Perf^+^, and GranzB^+^Perf^+^ in CD8^+^ T-cells in the spleen of WT and CD73KO mice at 14 dpi (n = 4 per group). **(E)** Frequencies of CD107a^+^ cells in CD8^+^ T-cells in spleens from WT and CD73KO mice at 14 dpi (n = 4) after *T. cruzi* lysate stimulation. Independent samples t-test was performed to compare WT vs. CD73KO. *p < 0.05, **p < 0.01, ***p < 0.001, ****p < 0.0001.

Supplementary Figure 2 CD4 T-cells contain higher expression of P2X7R than CD8 T-cells **(A)** t-SNE visualization of FACS data from splenic CD3^+^ cells (pre-gated on singlets, cells, and live cells) from WT mice at 14 dpi. t-SNE plots are color-coded according to expression levels of the indicated markers (red = highest expression; blue = lowest). Frequencies of CD4^+^CD8^-^, CD4^-^CD8^+^, and CD4^-^CD8^-^ in T-cells from WT mice at 14 dpi, and the expression of P2X7 in each subset. **(B)** LDH levels in the supernatants from co-cultures of splenic CD4^+^ T-cells from WT and CD73KO mice at 14 dpi with *in vitro* infected peritoneal macrophages (n = 3 per condition). A: Data was analyzed using a one-way ANOVA followed by Tukey´s *post hoc* multiple comparisons test. B: Independent samples t-test was performed to compare WT vs. CD73KO. **p < 0.01, ***p < 0.001, ****p < 0.0001.

Supplementary Figure 3 CD73 deficiency does not alter the abundance of cardiac CD8 CTLs **(A-D)** Frequencies and number of cells per milligram of tissue of CD8^+^ T-cells and GranzB^+^Perf^-^, GranzB^-^Perf^+^, and GranzB^+^Perf^+^ CD8^+^ T-cells in cardiac tissue from WT and CD73KO mice at 21 dpi (A, B) and 250 dpi (C, D) (n = 4 per group). Independent samples t-test was performed to compare WT vs. CD73KO. *p < 0.05.

Supplementary Figure 4 CD73KO mice exhibit cardiac dysfunction **(A)** Relative expressions of *Wnt3a* and *Wnt5a* in cardiac tissue of WT and CD73KO mice at 250 dpi (n = 3 - 4). mRNA gene expression levels were relativized to that in WT mice. **(B)** Representative ECG tracings from WT (left) and CD73KO (right) mice at 250 dpi. **(C)** Detailed list of ECG parameters in WT and CD73KO at 250 dpi and non-infected CD73KO mice. Independent samples t-test was performed to compare WT vs. CD73KO. *p < 0.05.

Supplementary Figure 5 CD4^+^ T-cells from patients with Chagas disease display enhanced activation capacity **(A)** Expression of CD44 in CD4^+^ and CD8^+^ T-cells from PBMCs of Control and Chagas groups after 72 h of stimulation (n = 7 per group). **(B, C)** Frequencies of CD39^+^CD73^-^, CD39^-^CD73^+^, and CD39^+^CD73^+^ cells in CD4^+^ **(B)** or CD8^+^ **(C)** T-cells from Control and Chagas groups (n = 7 per group). Mann-Whitney test was performed to compare Control vs. Chagas. *p < 0.05.

Supplementary Figure 6 CD73 enrichment in immune cardiac cells Enrichment of *NT5e* transcript across different leukocyte populations inferred from the deconvolution analysis in CCC group.

## References

[1] Beatty NL Hamer GL Moreno-Peniche B Mayes B Hamer SA . Chagas disease, an endemic disease in the United States. Emerg Infect Dis. (2025) 31:1691–7. doi: 10.3201/eid3109.241700. PMID: 40866797 PMC12407112

[2] Hidron A Vogenthaler N Santos-Preciado JI Rodriguez-Morales AJ Franco-Paredes C Rassi A . Cardiac involvement with parasitic infections. Clin Microbiol Rev. (2010) 23:324–49. doi: 10.1128/CMR.00054-09. PMID: 20375355 PMC2863361

[3] MaChado FS Dutra WO Esper L Gollob KJ Teixeira MM Factor SM . Current understanding of immunity to Trypanosoma cruzi infection and pathogenesis of Chagas disease. Semin Immunopathol. (2012) 34:753–70. doi: 10.1007/s00281-012-0351-7. PMID: 23076807 PMC3498515

[4] Kölliker-Frers RA Otero-Losada M Razzitte G Calvo M Carbajales J Capani F . Chagas cardiomyopathy: Role of sustained host-parasite interaction in systemic inflammatory burden. In: Chagas disease - basic investigations and challenges. London, UK: IntechOpen (2018). Available online at: https://www.intechopen.com/chapters/62321. In: Chagas Disease - Basic Investigations and Challenges [Internet]. IntechOpen; 2018 [cited 2025 Apr 14]. doi: 10.5772/intechopen.77980

[5] Cenerenti M Saillard M Romero P Jandus C . The era of cytotoxic CD4 T cells. Front Immunol. (2022) 13:867189. doi: 10.3389/fimmu.2022.867189. PMID: 35572552 PMC9094409

[6] Bergero G Mazzocco YL Del Rosso S Liu R Cejas Gallardo ZM Robson SC . Purinergic signaling modulates CD4+ T cells with cytotoxic potential during Trypanosoma cruzi infection. J Clin Invest. (2025) 135:e186785. doi: 10.1172/JCI186785. PMID: 40590226 PMC12208558

[7] Barbosa CHD Canto FB Gomes A Brandao LM Lima JR Melo GA . Cytotoxic CD4+ T cells driven by T-cell intrinsic IL-18R/MyD88 signaling predominantly infiltrate Trypanosoma cruzi-infected hearts. eLife. (2022) 11:e74636. doi: 10.7554/eLife.74636. PMID: 35670567 PMC9236613

[8] Ferreira LRP Ferreira FM Laugier L Cabantous S Navarro IC da Silva Cândido D . Integration of miRNA and gene expression profiles suggest a role for miRNAs in the pathobiological processes of acute Trypanosoma cruzi infection. Sci Rep. (2017) 7:17990. doi: 10.1038/s41598-017-18080-9. PMID: 29269773 PMC5740174

[9] Nunes MCP Beaton A Acquatella H Bern C Bolger AF Echeverría LE . Chagas cardiomyopathy: An update of current clinical knowledge and management: A scientific statement from the American Heart Association. Circulation. (2018) 138:e169–209. doi: 10.1161/CIR.0000000000000599. PMID: 30354432

[10] Takeuchi A Saito T . CD4 CTL, a cytotoxic subset of CD4+ T cells, their differentiation and function. Front Immunol. (2017) 8:194. doi: 10.3389/fimmu.2017.00194. PMID: 28280496 PMC5321676

[11] Khakh BS Burnstock G . The double life of ATP. Sci Am. (2009) 301:84–92. doi: 10.1038/scientificamerican1209-84. PMID: 20058644 PMC2877495

[12] Langer D Hammer K Koszalka P Schrader J Robson S Zimmermann H . Distribution of ectonucleotidases in the rodent brain revisited. Cell Tissue Res. (2008) 334:199–217. doi: 10.1007/s00441-008-0681-x. PMID: 18843508

[13] Antonioli L Pacher P Vizi ES Haskó G . CD39 and CD73 in immunity and inflammation. Trends Mol Med. (2013) 19:6. doi: 10.1016/j.molmed.2013.03.005. PMID: 23601906 PMC3674206

[14] Ponce NE Sanmarco LM Eberhardt N García MC Rivarola HW Cano RC . CD73 inhibition shifts cardiac macrophage polarization toward a microbicidal phenotype and ameliorates the outcome of experimental Chagas cardiomyopathy. J Immunol. (2016) 197:814–23. doi: 10.4049/jimmunol.1600371. PMID: 27335499

[15] Eberhardt N Sanmarco LM Bergero G Favaloro RR Vigliano C Aoki MP . HIF-1α and CD73 expression in cardiac leukocytes correlates with the severity of myocarditis in end-stage Chagas disease patients. Biochim Biophys Acta Mol Basis Dis. (2020) 1866:165592. doi: 10.1016/j.bbadis.2019.165592. PMID: 31678157

[16] S D Km D W G D F A U A E . Adenosine generation catalyzed by CD39 and CD73 expressed on regulatory T cells mediates immune suppression. J Exp Med. (2007) 204:1257–65. doi: 10.1084/jem.20062512. PMID: 17502665 PMC2118603

[17] Zhou Q Yan J Putheti P Wu Y Sun X Toxavidis V . Isolated CD39 expression on CD4+ T cells denotes both regulatory and memory populations. Am J Transplant. (2009) 9:2303–11. doi: 10.1111/j.1600-6143.2009.02777.x. PMID: 19656134 PMC2930268

[18] Raczkowski F Rissiek A Ricklefs I Heiss K Schumacher V Wundenberg K . CD39 is upregulated during activation of mouse and human T cells and attenuates the immune response to Listeria monocytogenes. PloS One. (2018) 13:e0197151. doi: 10.1371/journal.pone.0197151. PMID: 29742141 PMC5942830

[19] Duhen T Duhen R Montler R Moses J Moudgil T de Miranda NF . Co-expression of CD39 and CD103 identifies tumor-reactive CD8 T cells in human solid tumors. Nat Commun. (2018) 9:2724. doi: 10.1038/s41467-018-05072-0. PMID: 30006565 PMC6045647

[20] Eberhardt N Sanmarco LM Bergero G Favaloro RR Vigliano C Aoki MP . HIF-1α and CD73 expression in cardiac leukocytes correlates with the severity of myocarditis in end-stage Chagas disease patients. J Leukoc Biol. (2021) 109:1. doi: 10.1002/JLB.4MA0420-125R. PMID: 32450615

[21] Linden J Cekic C . Regulation of lymphocyte function by adenosine. Arterioscler Thromb Vasc Biol. (2012) 32:2097–103. doi: 10.1161/ATVBAHA.111.226837. PMID: 22772752 PMC4476649

[22] Takeuchi A Badr MESG Miyauchi K Ishihara C Onishi R Guo Z . CRTAM determines the CD4+ cytotoxic T lymphocyte lineage. J Exp Med. (2016) 213:123–38. doi: 10.1084/jem.20150519. PMID: 26694968 PMC4710199

[23] Marshall NB Swain SL . Cytotoxic CD4 T cells in antiviral immunity. J BioMed Biotechnol. (2011) 2011:954602. doi: 10.1155/2011/954602. PMID: 22174559 PMC3228492

[24] Tsukamoto H Senju S Matsumura K Swain SL Nishimura Y . IL-6-mediated environmental conditioning of defective Th1 differentiation dampens antitumour immune responses in old age. Nat Commun. (2015) 6:6702. doi: 10.1038/ncomms7702. PMID: 25850032 PMC4396369

[25] Devarajan P Vong AM Castonguay CH Silverstein NJ Kugler-Umana O Bautista BL . Cytotoxic CD4 development requires CD4 effectors to concurrently recognize local antigen and encounter type I IFN-induced IL-15. Cell Rep. (2023) 42:113182. doi: 10.1016/j.celrep.2023.113182. PMID: 37776519 PMC10842051

[26] Brown DM Kamperschroer C Dilzer AM Roberts DM Swain SL . IL-2 and antigen dose differentially regulate perforin- and FasL-mediated cytolytic activity in antigen specific CD4+ T cells. Cell Immunol. (2009) 257:69–79. doi: 10.1016/j.cellimm.2009.03.002. PMID: 19338979 PMC2683476

[27] Rassi A Marin JA Rassi A . Chronic Chagas cardiomyopathy: a review of the main pathogenic mechanisms and the efficacy of aetiological treatment following the BENznidazole Evaluation for Interrupting Trypanosomiasis (BENEFIT) trial. Mem Inst Oswaldo Cruz. (2017) 112:224–35. doi: 10.1590/0074-02760160334. PMID: 28225900 PMC5319366

[28] Działo E Czepiel M Tkacz K Siedlar M Kania G Błyszczuk P . WNT/β-catenin signaling promotes TGF-β-mediated activation of human cardiac fibroblasts by enhancing IL-11 production. Int J Mol Sci. (2021) 22:10072. doi: 10.3390/ijms221810072. PMID: 34576234 PMC8468519

[29] Menezes CAS Sullivan AK Falta MT Mack DG Freed BM Rocha MOC . Highly conserved CDR3 region in circulating CD4+Vβ5+ T cells may be associated with cytotoxic activity in Chagas disease. Clin Exp Immunol. (2012) 169:109–18. doi: 10.1111/j.1365-2249.2012.04608.x. PMID: 22774985 PMC3406370

[30] Grossman WJ Verbsky JW Tollefsen BL Kemper C Atkinson JP Ley TJ . Differential expression of granzymes A and B in human cytotoxic lymphocyte subsets and T regulatory cells. Blood. (2004) 104:2840–8. doi: 10.1182/blood-2004-03-0859. PMID: 15238416

[31] Sanmarco LM Eberhardt N Bergero G Quebrada Palacio LP Adami PM Visconti LM . Monocyte glycolysis determines CD8+ T cell functionality in human Chagas disease. JCI Insight. (2019) 4:e123490. doi: 10.1172/jci.insight.123490. PMID: 31479429 PMC6795286

[32] Maya JD Orellana M Ferreira J Kemmerling U López-Muñoz R Morello A . Chagas disease: Present status of pathogenic mechanisms and chemotherapy. Biol Res. (2010) 43:3. doi:/S0716-97602010000300009 doi: 10.4067/S0716-97602010000300009 21249304

[33] Brochet P Ianni BM Laugier L Frade AF Silva Nunes JP Teixeira PC . Epigenetic regulation of transcription factor binding motifs promotes Th1 response in Chagas disease cardiomyopathy. Front Immunol. (2022) 13:958200. doi: 10.3389/fimmu.2022.958200. PMID: 36072583 PMC9441916

[34] Pérez-Antón E Egui A Thomas MC Simón M Segovia M López MC . Immunological exhaustion and functional profile of CD8+ T lymphocytes as cellular biomarkers of therapeutic efficacy in chronic Chagas disease patients. Acta Trop. (2020) 202:105242. doi: 10.1016/j.actatropica.2019.105242. PMID: 31669531

[35] Eberhardt N Sanmarco LM Bergero G Theumer MG García MC Ponce NE . Deficiency of CD73 activity promotes protective cardiac immunity against Trypanosoma cruzi infection but permissive environment in visceral adipose tissue. Biochim Biophys Acta (BBA) - Mol Basis Dis. (2020) 1866:165592. doi: 10.1016/j.bbadis.2019.165592. PMID: 31678157

[36] Sanmarco LM Eberhardt N Ponce NE Cano RC Bonacci G Aoki MP . New insights into the immunobiology of mononuclear phagocytic cells and their relevance to the pathogenesis of cardiovascular diseases. Front Immunol. (2017) 8:1921. doi: 10.3389/fimmu.2017.01921. PMID: 29375564 PMC5767236

[37] Koshiba M Rosin DL Hayashi N Linden J Sitkovsky MV . Patterns of A2A extracellular adenosine receptor expression in different functional subsets of human peripheral T cells. Flow cytometry studies with anti-A2A receptor monoclonal antibodies. Mol Pharmacol. (1999) 55:614–24. doi: 10.1016/s0026-895x(24)12188-8. PMID: 10051547

[38] Rissiek B Haag F Boyer O Koch-Nolte F Adriouch S . P2X7 on mouse T cells: One channel, many functions. Front Immunol. (2015) 6:204. doi: 10.3389/fimmu.2015.00204. PMID: 26042119 PMC4436801

[39] Jacobson KA Gao ZG . Adenosine receptors as therapeutic targets. Nat Rev Drug Discov. (2006) 5:247–64. doi: 10.1038/nrd1983. PMID: 16518376 PMC3463109

[40] Burnstock G . The therapeutic potential of purinergic signalling. Biochem Pharmacol. (2018) 151:157–65. doi: 10.1016/j.bcp.2017.07.016. PMID: 28735873

[41] Delgobo M Heinrichs M Hapke N Ashour D Appel M Srivastava M . Terminally differentiated CD4+ T cells promote myocardial inflammaging. Front Immunol. (2021) 12:584538. doi: 10.3389/fimmu.2021.584538. PMID: 33679735 PMC7935504

[42] Broadley I Pera A Morrow G Davies KA Kern F . Expansions of cytotoxic CD4+CD28- T cells drive excess cardiovascular mortality in rheumatoid arthritis and other chronic inflammatory conditions and are triggered by CMV infection. Front Immunol. (2017) 8:195. doi: 10.3389/fimmu.2017.00195. PMID: 28303136 PMC5332470

[43] Araujo Furlan CL Tosello Boari J Rodriguez C Canale FP Fiocca Vernengo F Boccardo S . Limited Foxp3+ regulatory T cells response during acute Trypanosoma cruzi infection is required to allow the emergence of robust parasite-specific CD8+ T cell immunity. Front Immunol. (2018) 9:2555. doi: 10.3389/fimmu.2018.02555. PMID: 30455700 PMC6230662

[44] Eckle T Krahn T Grenz A Köhler D Mittelbronn M Ledent C . Cardioprotection by ecto-5′-nucleotidase (CD73) and A2B adenosine receptors. Circulation. (2007) 115:1581–90. doi: 10.1161/CIRCULATIONAHA.106.669697. PMID: 17353435

[45] Ndzie Noah ML Mprah R Wowui PI Adekunle AO Adu-Amankwaah J Tan R . CD73/adenosine axis exerts cardioprotection against hypobaric hypoxia-induced metabolic shift and myocarditis in a sex-dependent manner. Cell Commun Signal. (2024) 22:166. doi: 10.1186/s12964-024-01535-8. PMID: 38454449 PMC10918954

[46] Quast C Alter C Ding Z Borg N Schrader J . Adenosine formed by CD73 on T cells inhibits cardiac inflammation and fibrosis and preserves contractile function in transverse aortic constriction-induced heart failure. Circ Heart Fail. (2017) 10:e003346. doi: 10.1161/CIRCHEARTFAILURE.116.003346. PMID: 28404626

[47] Zhou Y Schneider DJ Blackburn MR . Adenosine signaling and the regulation of chronic lung disease. Pharmacol Ther. (2009) 123:105–16. doi: 10.1016/j.pharmthera.2009.04.003. PMID: 19426761 PMC2743314

[48] Zhong H Belardinelli L Zeng D . Pro-fibrotic role of the A2B adenosine receptor in human cardiac fibroblasts. J Cardiac Failure. (2011) 17:S65. doi: 10.1016/j.cardfail.2011.06.234. PMID: 38826717

[49] Higuchi ML De Brito T Martins Reis M Barbosa A Bellotti G Pereira-Barreto AC . Correlation between Trypanosoma cruzi parasitism and myocardial inflammatory infiltrate in human chronic chagasic myocarditis: Light microscopy and immunohistochemical findings. Cardiovasc Pathol. (1993) 2:101–6. doi: 10.1016/1054-8807(93)90021-S. PMID: 25990604

[50] Bellotti G Bocchi EA de Moraes AV Higuchi ML Barbero-Marcial M Sosa E . *In vivo* detection of Trypanosoma cruzi antigens in hearts of patients with chronic Chagas’ heart disease. Am Heart J. (1996) 131:301–7. doi: 10.1016/s0002-8703(96)90358-0. PMID: 8579025

[51] Schijman AG Vigliano CA Viotti RJ Burgos JM Brandariz S Lococo BE . Trypanosoma cruzi DNA in cardiac lesions of Argentinean patients with end-stage chronic chagas heart disease. Am J Trop Med Hyg. (2004) 70:210–20. doi: 10.4269/ajtmh.2004.70.210. PMID: 14993635

[52] Burgos JM Diez M Vigliano C Bisio M Risso M Duffy T . Molecular identification of Trypanosoma cruzi discrete typing units in end-stage chronic Chagas heart disease and reactivation after heart transplantation. Clin Infect Dis. (2010) 51:485–95. doi: 10.1086/655680. PMID: 20645859

[53] Maravelia P Yao H Cai C Nascimento Silva D Fransson J Nilsson OB . Unlocking novel T cell-based immunotherapy for hepatocellular carcinoma through neoantigen-driven T cell receptor isolation. Gut. (2025) 74:gutjnl-2024-334148. doi: 10.1136/gutjnl-2024-334148. PMID: 39832892 PMC12611799

[54] Borgers JSW Lenkala D Kohler V Jackson EK Linssen MD Hymson S . Personalized, autologous neoantigen-specific T cell therapy in metastatic melanoma: a phase 1 trial. Nat Med. (2025) 31:881–93. doi: 10.1038/s41591-024-03418-4. PMID: 39753970 PMC11922764

[55] Sanmarco LM Ponce NE Visconti LM Eberhardt N Theumer MG Minguez ÁR . IL-6 promotes M2 macrophage polarization by modulating purinergic signaling and regulates the lethal release of nitric oxide during Trypanosoma cruzi infection. Biochim Biophys Acta Mol Basis Dis. (2017) 1863:857–69. doi: 10.1016/j.bbadis.2017.01.006. PMID: 28087471

[56] Ponce NE Cano RC Carrera-Silva EA Lima AP Gea S Aoki MP . Toll-like receptor-2 and interleukin-6 mediate cardiomyocyte protection from apoptosis during Trypanosoma cruzi murine infection. Med Microbiol Immunol. (2012) 201:145–55. doi: 10.1007/s00430-011-0216-z. PMID: 21984337

[57] Piron M Fisa R Casamitjana N López-Chejade P Puig L Vergés M . Development of a real-time PCR assay for Trypanosoma cruzi detection in blood samples. Acta Trop. (2007) 103:195–200. doi: 10.1016/j.actatropica.2007.05.019. PMID: 17662227

