# Supplementary figures and images for: CD73 activity controls cytotoxic CD4 T-cell response driving myocardial pathology in chronic Chagas disease
Source: Front Immunol. 2026 May 12;17:1770276. doi: 10.3389/fimmu.2026.1770276 (PMC13201142; doi:10.3389/fimmu.2026.1770276)

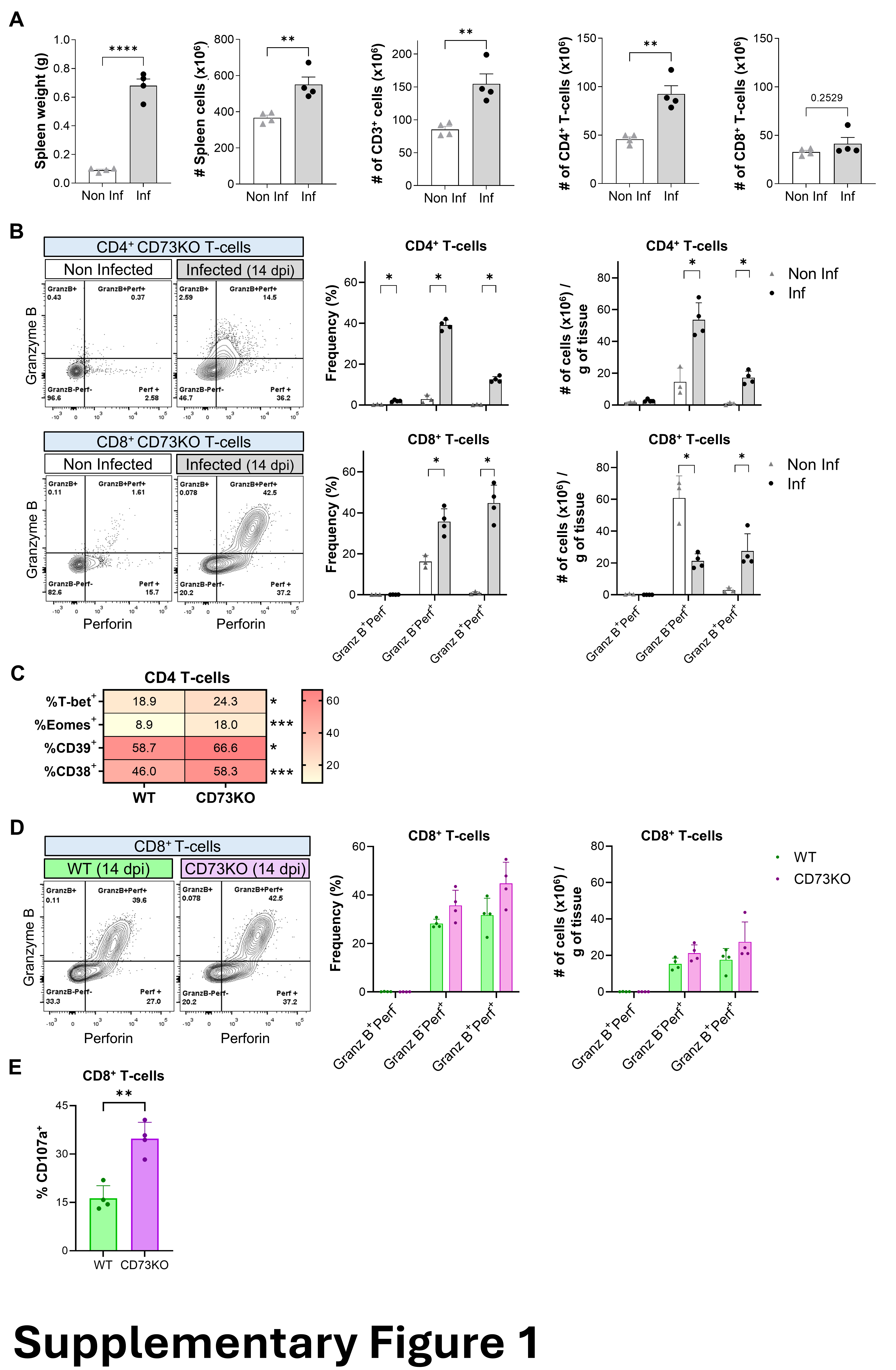

Supplement: Supplementary Figure 1 — CD73 deficiency no expand the CD8 CTL population (A) Spleen weights and total numbers of splenic cells, CD3+ cells (T-cells), CD4+ and CD8+ T-cells from non-infected CD73KO mice (Non Inf, n = 4) and at 14 dpi (Inf, n = 4). (B) Representative contour plots, frequencies and number of cells per gram of tissue of GranzB+Perf-, GranzB-Perf+, and GranzB+Perf+ cells in CD4+ or CD8+ T-cells in non-infected CD73KO mice and at 14 dpi. (C) Heatmap comparing the frequencies of CD4+ T-cells expressing T-bet, Eomes, CD39, and CD38 between WT and CD73KO mice at 14 dpi (n = 4 per group). (D) Representative contour plots, frequencies and number of cells per gram of tissue of GranzB+Perf-, GranzB-Perf+, and GranzB+Perf+ in CD8+ T-cells in the spleen of WT and CD73KO mice at 14 dpi (n = 4 per group). (E) Frequencies of CD107a+ cells in CD8+ T-cells in spleens from WT and CD73KO mice at 14 dpi (n = 4) after T. cruzi lysate stimulation. Independent samples t-test was performed to compare WT vs. CD73KO. *p < 0.05, **p < 0.01, ***p < 0.001, ****p < 0.0001. [file Image1.tif]

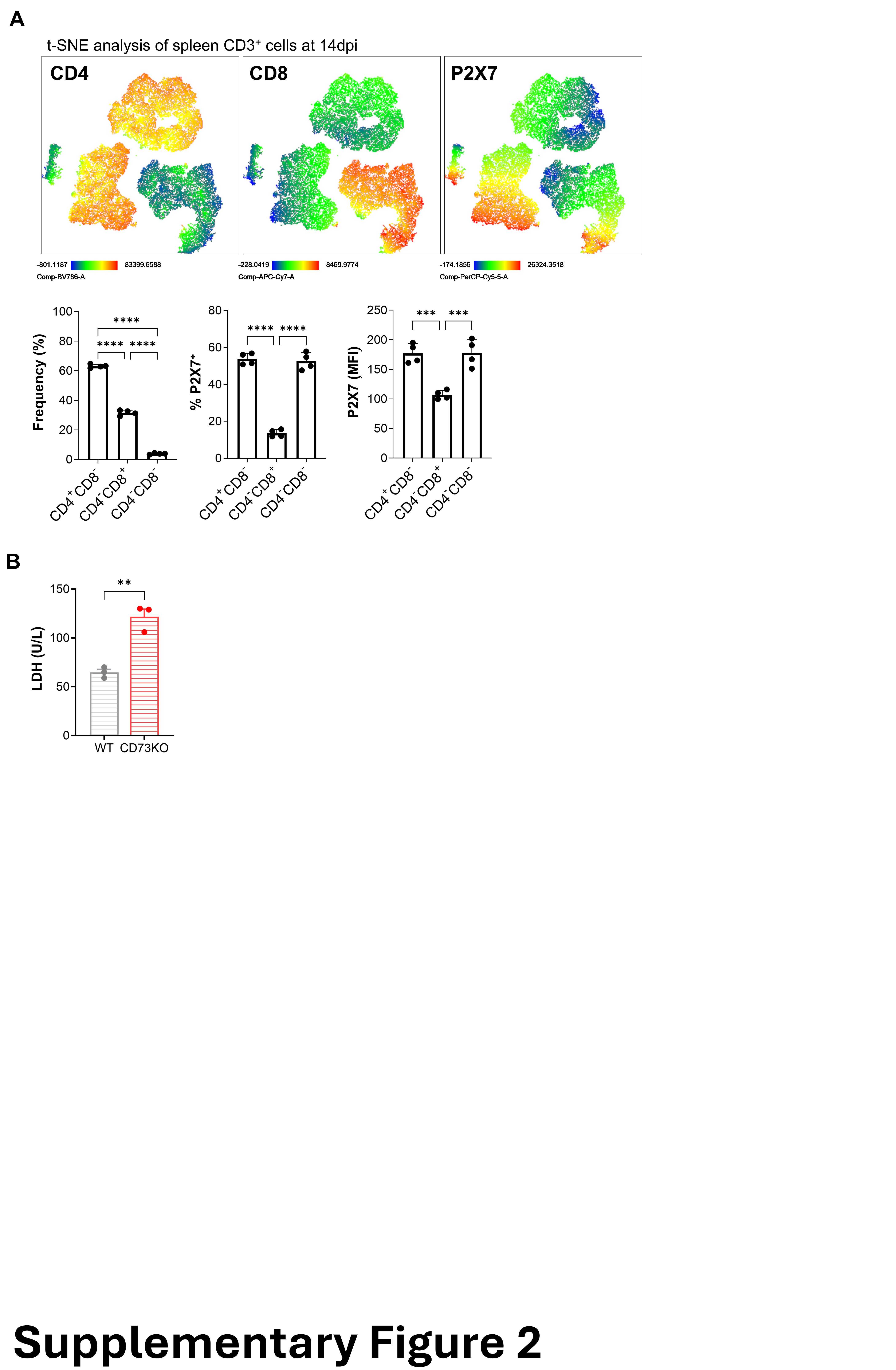

Supplement: Supplementary Figure 2 — CD4 T-cells contain higher expression of P2X7R than CD8 T-cells (A) t-SNE visualization of FACS data from splenic CD3+ cells (pre-gated on singlets, cells, and live cells) from WT mice at 14 dpi. t-SNE plots are color-coded according to expression levels of the indicated markers (red = highest expression; blue = lowest). Frequencies of CD4+CD8-, CD4-CD8+, and CD4-CD8- in T-cells from WT mice at 14 dpi, and the expression of P2X7 in each subset. (B) LDH levels in the supernatants from co-cultures of splenic CD4+ T-cells from WT and CD73KO mice at 14 dpi with in vitro infected peritoneal macrophages (n = 3 per condition). A: Data was analyzed using a one-way ANOVA followed by Tukey´s post hoc multiple comparisons test. B: Independent samples t-test was performed to compare WT vs. CD73KO. **p < 0.01, ***p < 0.001, ****p < 0.0001. [file Image2.tif]

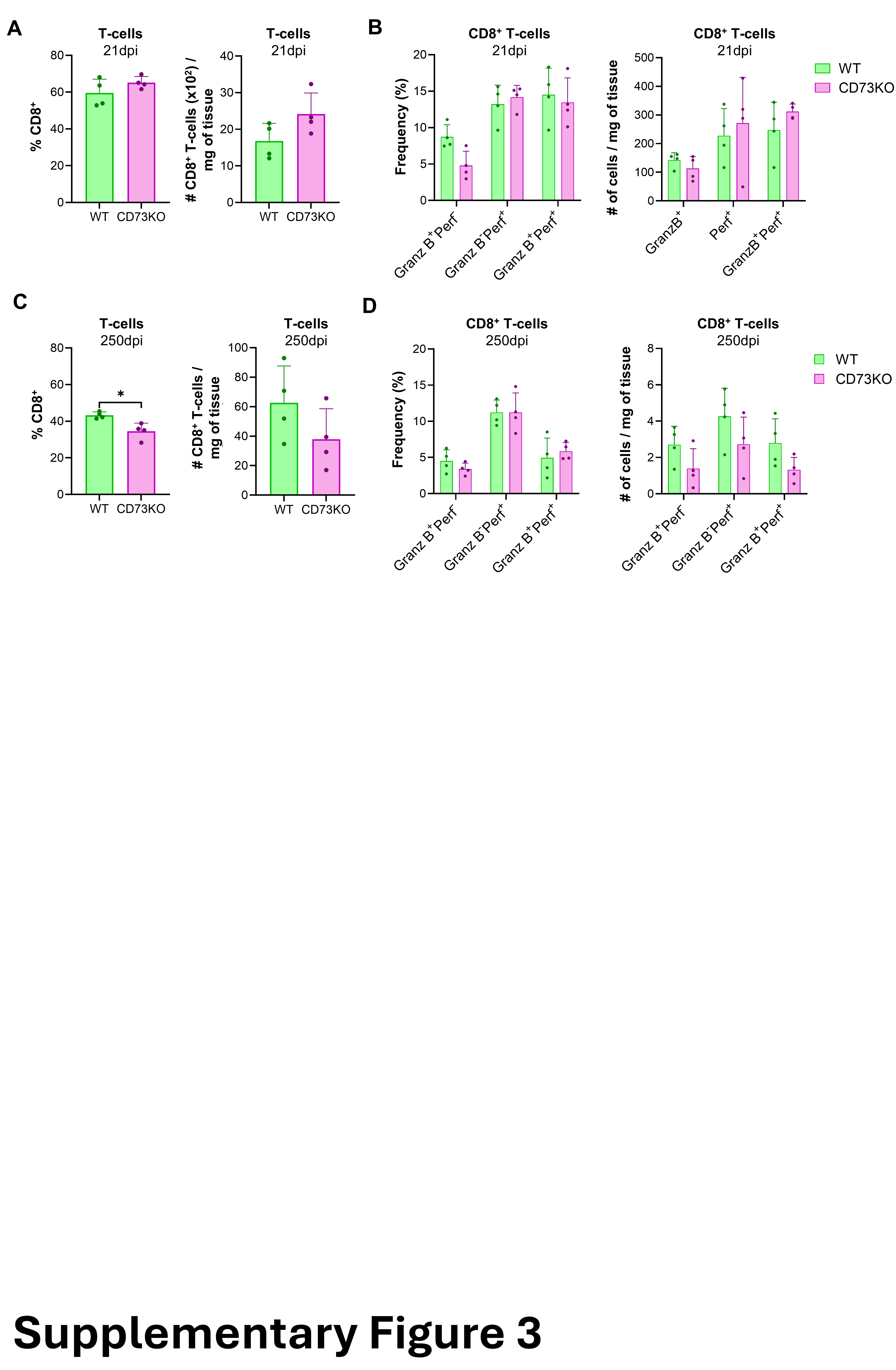

Supplement: Supplementary Figure 3 — CD73 deficiency does not alter the abundance of cardiac CD8 CTLs (A-D) Frequencies and number of cells per milligram of tissue of CD8+ T-cells and GranzB+Perf-, GranzB-Perf+, and GranzB+Perf+ CD8+ T-cells in cardiac tissue from WT and CD73KO mice at 21 dpi (A, B) and 250 dpi (C, D) (n = 4 per group). Independent samples t-test was performed to compare WT vs. CD73KO. *p < 0.05. [file Image3.tif]

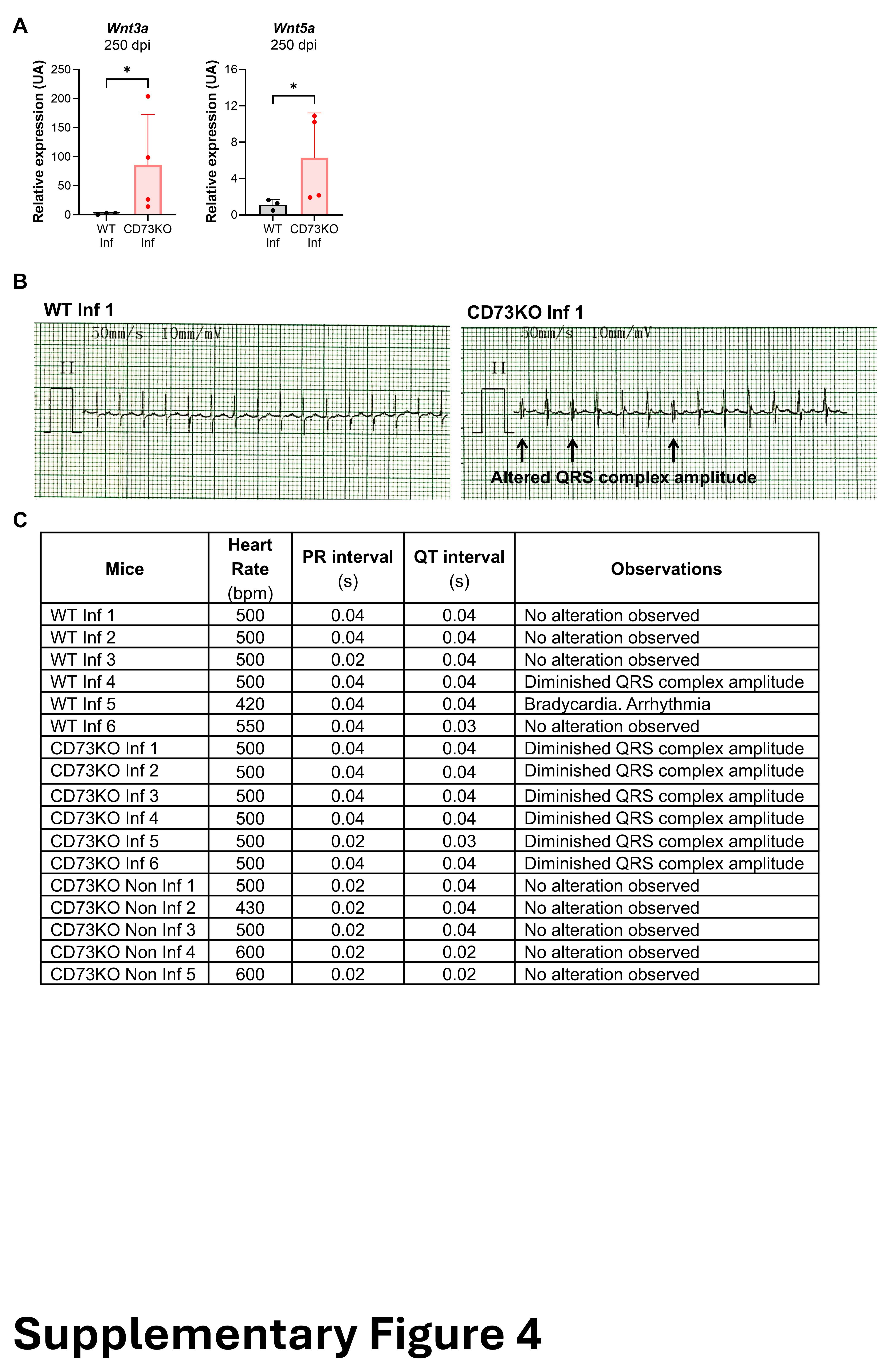

Supplement: Supplementary Figure 4 — CD73KO mice exhibit cardiac dysfunction (A) Relative expressions of Wnt3a and Wnt5a in cardiac tissue of WT and CD73KO mice at 250 dpi (n = 3 - 4). mRNA gene expression levels were relativized to that in WT mice. (B) Representative ECG tracings from WT (left) and CD73KO (right) mice at 250 dpi. (C) Detailed list of ECG parameters in WT and CD73KO at 250 dpi and non-infected CD73KO mice. Independent samples t-test was performed to compare WT vs. CD73KO. *p < 0.05. [file Image4.tif]

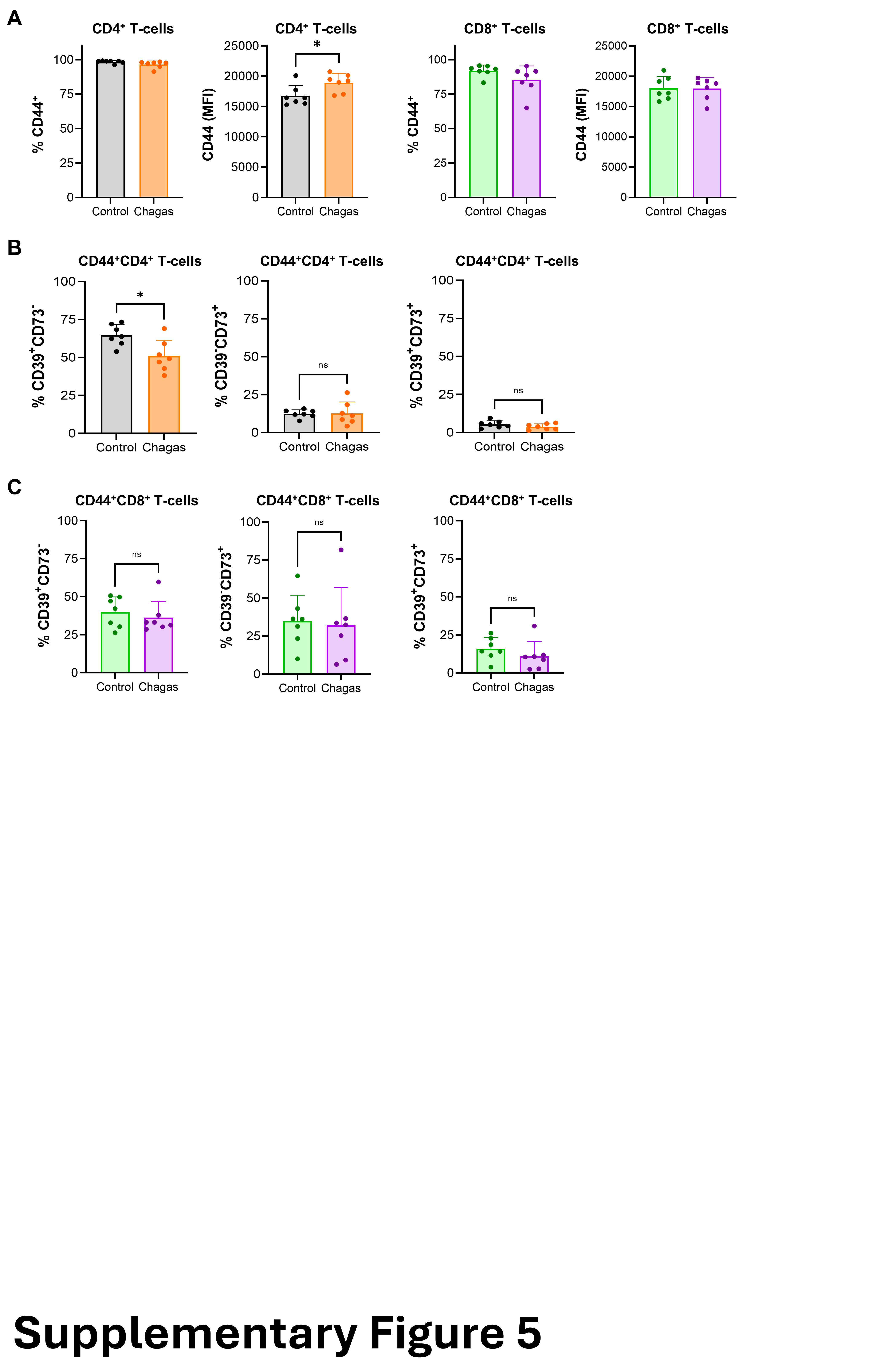

Supplement: Supplementary Figure 5 — CD4+ T-cells from patients with Chagas disease display enhanced activation capacity (A) Expression of CD44 in CD4+ and CD8+ T-cells from PBMCs of Control and Chagas groups after 72 h of stimulation (n = 7 per group). (B, C) Frequencies of CD39+CD73-, CD39-CD73+, and CD39+CD73+ cells in CD4+ (B) or CD8+ (C) T-cells from Control and Chagas groups (n = 7 per group). Mann-Whitney test was performed to compare Control vs. Chagas. *p < 0.05. [file Image5.tif]

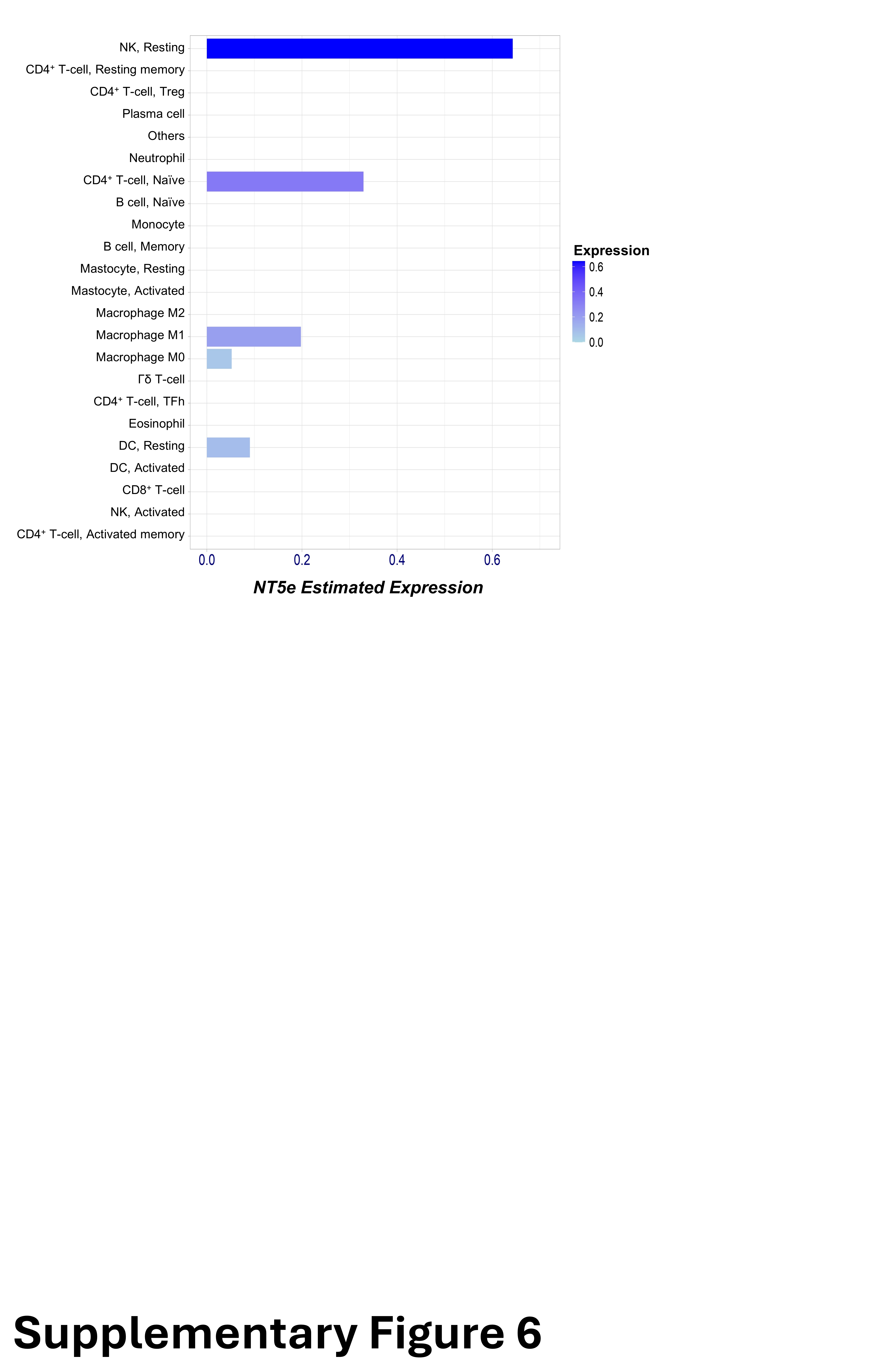

Supplement: Supplementary Figure 6 — CD73 enrichment in immune cardiac cells Enrichment of NT5e transcript across different leukocyte populations inferred from the deconvolution analysis in CCC group. [file Image6.tif]

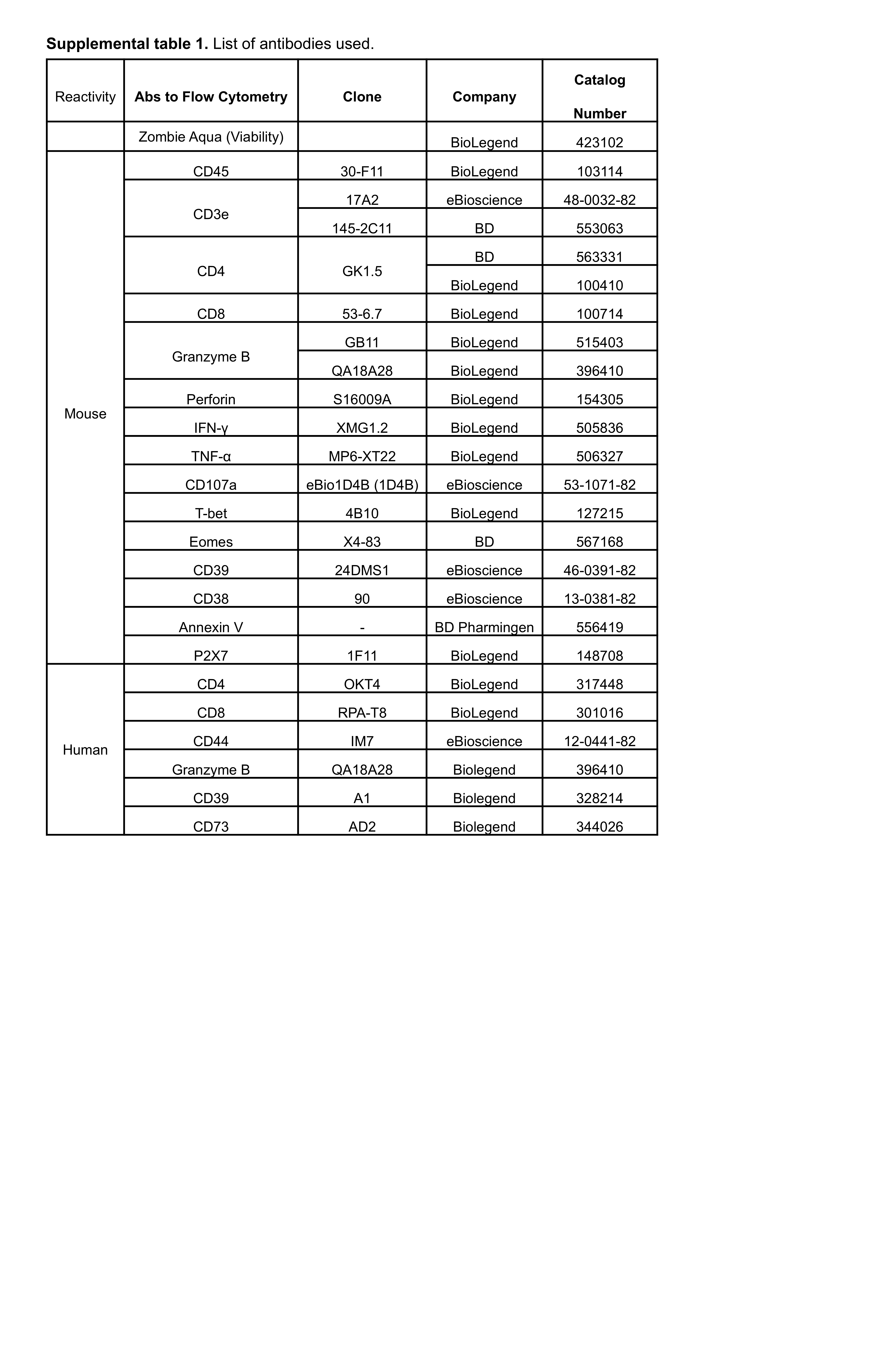

Supplement: Supplementary file 7 [file Image7.tif]
